# Metabolomic and elemental profiling of human tissue in kidney cancer

**DOI:** 10.1007/s11306-021-01779-2

**Published:** 2021-03-04

**Authors:** Joanna Nizioł, Valérie Copié, Brian P. Tripet, Leonardo B. Nogueira, Katiane O. P. C. Nogueira, Krzysztof Ossoliński, Adrian Arendowski, Tomasz Ruman

**Affiliations:** 1 Faculty of Chemistry, Rzeszów University of Technology, 6 Powstańców Warszawy Ave., 35-959 Rzeszów, Poland; 2 The Department of Chemistry and Biochemistry, Montana State University, Bozeman, MT 59717 USA; 3 Department of Geology, Federal University of Ouro Preto, Ouro Preto, Minas Gerais Brazil; 4 Department of Biological Sciences, Federal University of Ouro Preto, Ouro Preto, Minas Gerais Brazil; 5 Department of Urology, John Paul II Hospital, Grunwaldzka 4 St., 36-100 Kolbuszowa, Poland

**Keywords:** Human kidney tissue, Cancer, Biomarkers, Metallomics, Metabolomics

## Abstract

**Introduction:**

Kidney cancer is one of the most frequently diagnosed and the most lethal urinary cancer. Despite advances in treatment, no specific biomarker is currently in use to guide therapeutic interventions.

**Objectives:**

Major aim of this work was to perform metabolomic and elemental profiling of human kidney cancer and normal tissue and to evaluate cancer biomarkers.

**Methods:**

Metabolic and elemental profiling of tumor and adjacent normal human kidney tissue from 50 patients with kidney cancer was undertaken using three different analytical methods.

**Results:**

Five potential tissue biomarkers of kidney cancer were identified and quantified using with high-resolution nuclear magnetic resonance spectroscopy. The contents of selected chemical elements in tissues was analyzed using inductively coupled plasma optical emission spectrometry. Eleven mass spectral features differentiating between kidney cancer and normal tissues were detected using silver-109 nanoparticle enhanced steel target laser desorption/ionization mass spectrometry.

**Conclusions:**

Our results, derived from the combination of ICP-OES, LDI MS and 1H NMR methods, suggest that tissue biomarkers identified herein appeared to have great potential for use in clinical prognosis and/or diagnosis of kidney cancer.

**Supplementary Information:**

The online version contains supplementary material available at 10.1007/s11306-021-01779-2.

## Introduction

Kidney cancer is one of the most frequently diagnosed metabolic diseases of the urinary tract. More than 400 thousand new cases of kidney cancer and nearly 180 thousand deaths occurred in 2018 (Bray et al., 2018). Based on histological classification, a number of different types of kidney cancers were classified including both benign tumors like adenoma, oncocytoma and angiomyolipoma (AML) and the most common malignant type of kidney cancer, renal cell carcinoma (RCC). RCC is accounting for approximately ninety percent of all neoplasms originating from the kidney. There are three main types of RCC known, namely clear cell RCC (ccRCC), papillary RCC (pRCC) and chromophobe RCC (cRCC). Mentioned types may differ in stage, grade, and cancer-specific survival. Other subtypes of RCC are very rare and include angiomyolipoma (AML), collecting duct carcinoma (CDC), or simple renal cyst (SRC) (Hsieh et al., 2017).

Currently, RCC diagnosis is based on magnetic resonance imaging, ultrasound examination or computed tomography. Unfortunately, more than 60% of RCC cases are diagnosed incidentally. This tumor is difficult to detect, especially in its early stages, due to the lack of characteristic symptoms including lack of the classic triad of visible haematuria, flank pain and palpable abdominal mass symptoms. Poor prognosis and high mortality rate are related to metastases and resistance to chemotherapy and radiotherapy. The 5-year survival rates of patients with metastatic disease are less than ten percent (Lim et al., 2013). In spite of great efforts there are still no clinically available biomarkers for early detection, diagnosis or prognosis of kidney cancers. Analysis of metabolic profiles from tissues and biofluids is a promising approach for the discovery of biomarkers that would enhance our abilities to predict cancer progression and to assess the effectiveness of cancer treatment (Gupta et al., 2020).

Over the past two decades, two major analytical platforms have been employed for metabolomic analysis of kidney cancer samples of various types: (i) mass spectrometry (MS) (Lin et al., 2012) and (ii) nuclear magnetic resonance (NMR) (Gao et al., 2012). Tissues from patients with kidney cancer have been studied using primarily mass spectrometry (Gupta et al., 2020). The Catchpole et al. was one of the first to use profiling of low molecular weight compounds, such as sugars, lipids and amino acids to characterize the metabolic signature of RCC (Catchpole et al., 2011). Later, various groups performed focused profiling studies based mainly on LC- and GC–MS approaches (Shim et al., 2014; Wettersten et al., 2015). Interestingly, there exist no published studies of laser mass spectrometry data of human tissue extracts to date.

Nuclear magnetic resonance-based metabolomic studies of RCC tissue samples are relatively rarely found in literature (Gao et al., 2008). Previously, ^1^H magic angle spinning (MAS) NMR was used to analyze kidney carcinoma biopsy samples (Moka et al., 1998; Tate et al., 2000). To our knowledge, there is only one publication concerning the utilization of ^1^H NMR in the metabolomic analysis of human RCC tissues—in 2012 Gao et al. applied ^1^H NMR to the characterization of RCC metastases in tissue extracts (Gao et al., 2012). No published studies that combine both NMR, LDI MS obtained from tissues of patients with kidney cancer have been reported to date.

Many studies have suggested a relationship between toxic metals and trace elements (TMTE) and the development of carcinoma in humans (Mulware, 2013). Toxic elements can cause genetic and epigenetic effects that may result in increased risk of different cancer types (Mishra et al., 2014). ICP-OES is one the most frequently used approaches to determine metal elements and speciation in biological samples, and has been employed to assess the effects of TMTE on kidney tissue samples from patients with RCC (Abdel-Gawad et al., 2020). The results showed that the differences in the concentration of TMTE between normal and malignant tissue could be used as a biomarker of this disease.

In this study, we performed the first targeted and untargeted metabolomic and metallomic profiling of 100 human tissue samples using three different analytical platforms: NMR, ICP-OES and LDI MS. The value of laser desorption/ionization MS using a 109-silver nanoparticle-enhanced steel target (^109^AgNPET LDI MS) approach for metabolomics has been demonstrated in the detection of metabolites in plant and human tissues (Arendowski et al., 2018; Nizioł et al., 2019, Nizioł, Ossoliński, et al., 2020, 2021; Nizioł, Sunner, et al., 2020).

## Materials and methods

### Materials and equipment

^109^AgNPET was prepared as described previously (Nizioł et al., 2013). 2,5-Dihydroxybenzoic acid (DHB) was purchased from Aldrich. Silver-109 was purchased from BuyIsotope (Sweden). All solvents were of minimum ‘HPLC purity’, except for methanol and water (LCMS grade, Fluka) Deuterium oxide (D_2_O) and 4,4-dimethyl-4-silapentane-1-sulfonic acid (DSS) NMR reagents were purchased from Sigma Inc. Nitric acid 65% p.a EMSURE ISO and Hydrogen peroxide 30% p.a. EMSURE ACS ISO was purchased from Merck.

### Collection of tissue samples

Tissue samples were collected from 50 kidney cancer patients (20 females, 30 males, average age 69) undergoing surgical treatment, following detailed clinical questioning at John Paul II Hospital in Kolbuszowa (Poland). The study protocol was approved by local Bioethics Committee at the University of Rzeszow (Poland, permission no. 2018/04/10). All the patients in this study were of Caucasian race. All research was performed in accordance with relevant guidelines and regulations. Specimens and clinical data from patients involved in the study were collected with informed consent. All laboratory test results (complete blood count, urine analysis, bleeding profile, kidney function tests, CRP) were within normal ranges. Each patient donated 1 cm^3^ of renal cancer tissue (‘cancer’) dissected together with a small fragment of normal tissue (< 1 cm^3^, ‘controls’) removed ex vivo during radical nephrectomy. Samples were immediately frozen and stored at − 60 °C until further use. The pathological and clinical characteristics of the patients are presented in supplementary material table (Table S1).

### Preparation of tissue extracts for NMR and LDI MS studies

A weighted amount (10–168 mg) of sectioned tissue was transferred to a 2 ml centrifuge tube, then 900 µl of a 1:2 MeOH/CHCl_3_ (1:2, v/v) solution was added and homogenized with glass beads for 45 s. Next, a volume of 120 µl of cold H_2_O was added to each tube and then again homogenized two times for 45 s with a 5-min break time. The samples were placed at − 20 °C for 1 h and then centrifuged at 14,000 × *g* for 10 min at 4 °C to remove cells and other precipitated material. The polar (upper) phase was transferred to new 1.5 ml microcentrifuge tube, similarly non-polar (lower) phase was transferred to new 1.5 ml microcentrifuge tube. Finally, from all resulting samples, 50 µl volumes were taken and used for ^109^AgNPET LDI MS analyses. The rest of the sample was lyophilized to complete dryness using a SpeedVac vacuum concentrator (*ca.* 1 mbar), with no heat. Dried material was re-suspended in 600 µL of NMR buffer consisting of 25 mM NaH_2_PO_4_/Na_2_HPO_4_, 0.4 mM imidazole, 0.25 mM 4,4-dimethyl-4-silapentane-1-sulfonic acid (DSS) in 90% H_2_O/10% D_2_O, pH 7.0. Following re-suspension, samples were centrifuged at 21,000 rpm for 1 min to pellet insoluble debris, and then transferred to 5 mm NMR tubes for NMR metabolomics analysis.

### NMR spectra acquisition and data processing

NMR spectra acquisition, preprocessing and postprocessing were conducted as described recently (Nizioł, Ossoliński, et al., 2020; Nizioł, Sunner, et al., 2020). NMR data sets were normalized by tissue weight.

### MS spectra acquisition and preprocessing

Volume of 0.3 µl of each sample was placed on a ^109^AgNPET target plate and allowed to evaporate to dryness at room temperature, then target was inserted into a MALDI-type ToF/ToF mass spectrometer. Laser desorption ionization mass spectrometry (LDI MS) experiments were performed with a Bruker Autoflex Speed ToF mass spectrometer in positive-ion reflectron mode. The apparatus was using a SmartBeam II 355 nm laser. Laser impulse energy was approximately 100–190 μJ, laser repetition rate of 1000 Hz. Deflection was set on *m/z* 80, measurements were made within *m/z* range of 80–2000 Da. Spot of each extract was ablated by 20 k laser shots with a default random walk applied. All spectra were calibrated with silver ions: ^109^Ag^+^ to ^109^Ag_10_^+^. The first ion source voltage was held at 19 kV, and the second 16.7 kV. Reflector voltages were 21 kV (first) and 9.55 kV (second). MS data sets were normalized by tissue weight. FlexAnalysis 4.0 program was used for data processing and analysis.

### ICP-OES analysis

In order to determine the concentrations of major elements (Ca (calcium), Fe (iron), K (potassium), Na (sodium), Mg (magnesium)), minor elements (Mn (manganese),P (phosphorus), S (sulfur)) and trace elements (Cu (copper), and Zn (zinc)) in kidney cancer and normal tissue, 58 samples were taken for analysis with ICP-OES. All of the 58 samples were separated into three different groups according to similar mass. An analytical balance was used to verify sample mass. A 5 ml final solution volume was collected for all samples except for ten samples which had the highest mass; these resulted in a volume of 10 ml. The samples were weighted and placed in Teflon tubes (Savillex). 4 ml of HNO_3_ and 1 mL of H_2_O_2_ were added to the highest mass samples, while 3 ml of HNO_3_ and 0.5 ml of H_2_O_2_ were added to all other samples. The teflon tubes were closed and placed on the heating plate at 100 °C for 24 h. Following 24 h of digestion, ultrapure water was added to achieve the stated final volume of 5 ml. All Teflon tubes were weighted using an analytical balance and masses recorded. The final solutions were transferred to labeled flasks that had been washed with ultrapure water. The international standard NIST 1577c (bovine liver—NIST) was used between samples to monitor the instrumental performance and for quality control. The major, minor and trace elements were analyzed using the Agilent 725 Inductively Coupled Plasma Optical Emission Spectrometer. ICP-OES parameters and associated analytical conditions are reported in Table S2 (Supplementary information).

### Multivariate statistical analysis

Metabolite data was analyzed with the use of MetaboAnalyst software 4.0 (Chong et al., 2018). Data was log-transformed and auto-scaled prior to statistical analysis. Firstly, a total of 99 ^1^H NMR spectra were recorded on metabolite mixtures extracted from tissue samples of kidney cancer patients (49 tumor tissues and 50 adjacent normal tissues), 100 LDI MS spectra were recorded for the same 50 patients and 50 ICP-OES spectra were recorded for 58 kidney cancer patients (28 tumor tissues and 28 adjacent normal tissues). Resulting metabolite profiles from NMR, and MS and ICP-OES data sets were then subjected to unsupervised Principal Component Analysis (PCA). The separation between these two groups was examined Orthogonal Partial Least Squares Discriminant Analysis (OPLS-DA). The overall quality of the OPLS-DA models was assessed by examining the goodness of fit (R^2^Y) and the predictive ability of the models (Q^2^). S-plots were generated to identify metabolites whose level changes were most significantly responsible for groups separation. Metabolites with |p|> 0.05 (magnitude) and |p(corr)|> 0.5 (reliability) were considered as potential biomarker candidates to distinguish kidney cancer tissues from normal controls. To test the accuracy of the multivariate statistical models, and to rule out that the observed separation in the OPLS-DA is due to chance (p < 0.05), permutation tests were performed with 2000-fold repetition. Statistical significance of metabolite level differences was assessed with paired parametric t-test with Mann–Witney and Bonferroni correction. P-values and false discovery rates (FDR; q-value) less than 0.05 were considered statistically significant. In addition, receiver operating curve (ROC) analyses with random forest algorithm were undertaken to evaluate the diagnostic value of all and selected metabolites, elements and mass features for all models. Chemometric tools such as 2D PCA and OPLS-DA were also used to assess metabolic profile similarities and differences between malignant types of kidney cancer (ccRR, chRCC, pRCC, CDC, SRC) and benign (oncocytoma and AML). The relevant metabolic changes associated with age and gender were identified by one-way analysis of variance (ANOVA). To identify the most relevant metabolic pathways involved with kidney cancer, the metabolic pathway analysis was applied with MetaboAnalyst version 5.0 based on the Kyoto Encyclopedia of Genes and Genomes (KEGG) pathway library for *Homo sapiens*. Data was normalized with log-transformation and autoscaling. Enrichment analysis has been computed with global test. Topology analysis was based on Relative-betweenness Centrality. Quantitative enrichment analysis was conducted based on Small Molecule Pathway Database (SMPD). Each pathway was classified according to statistical p-value, Holm p (p-value adjusted by Holm–Bonferroni method) and FDR (p-value adjusted using False Discovery Rate), calculated from pathway topology analysis.

## Results

### Distinguishing between kidney cancer and control samples by ^1^H NMR

**Fig. 1:**
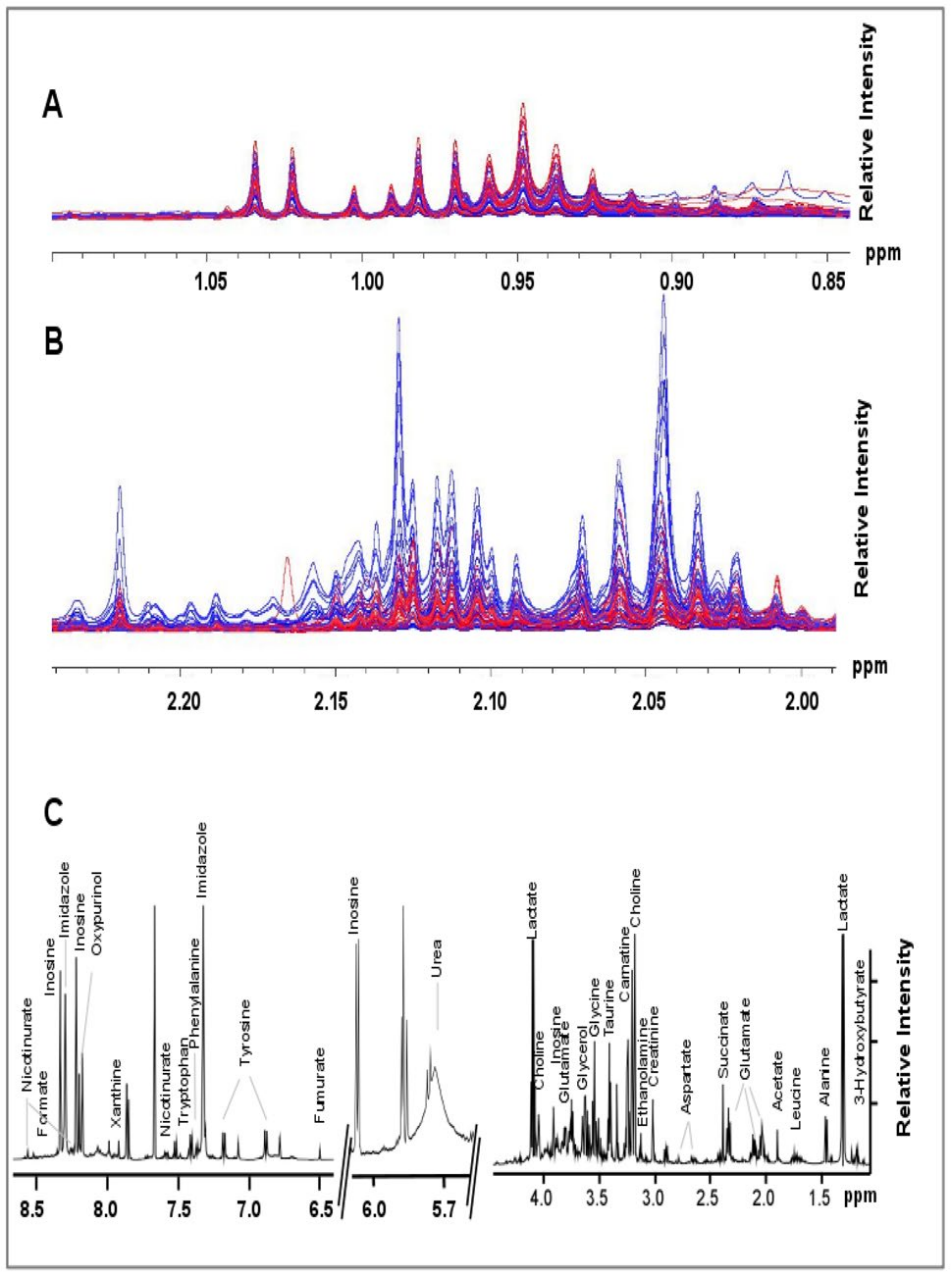
Representative 1D ^1^H NMR spectrum obtained from protein-free, polar metabolite extracts of kidney tissue obtained from control and kidney cancer patients. **a** Expanded spectral region spanning the ^1^H chemical shift range of 0.85 ppm to 1.10 ppm, depicting the overlay of 50 NMR spectra obtained from healthy (blue traces) and cancer (red traces) kidney tissue, and illustrating no obvious spectral difference between different sample types. **b** Expanded spectral region spanning the ^1^H chemical shift range of 2.00 ppm to 2.25 ppm, depicting the overlay of 50 NMR spectra obtained from healthy (blue traces) and cancer (red traces) kidney tissue, and illustrating obvious spectral difference between different sample types. **c** Characteristic 1D ^1^H NMR spectrum of protein-free, polar metabolite mixtures extracted from kidney cancer tissue and spanning the ^1^H chemical shift range of ~ 1 to 8.5 ppm, with characteristic signals arising from specific metabolites labeled

98 metabolite extracts from frozen kidney tissue samples (49 cancer and 49 control) were analyzed using high-resolution 1D ^1^H NMR to identify potential discriminant biomarkers of kidney cancer. In total, 48 metabolites were identified and quantified in each tissue sample using ^1^H NMR spectroscopy and metabolite profiling using Chenomx. Figure 1 presents a representative overlay of control and kidney cancer patient NMR spectra (blue and red traces, respectively) showing similarity and differences in the raw spectral data (plots A and B). Visual comparison of the NMR spectra revealed significant differences in individual metabolite levels between paired tissues.

**Fig. 2:**
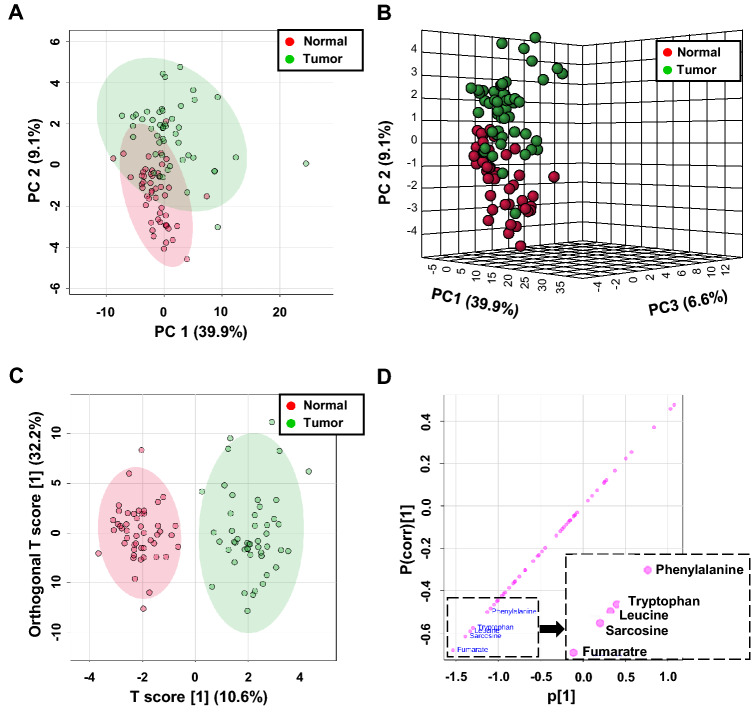
Analysis of tissue metabolite profiles created for ^1^H NMR data: **a** 2D PCA, **b** 3D PCA and **c** OPLS-DA scores plots of the tumor (red) and normal (green) tissue samples. **d** The OPLS-DA loading S-plot showing the distribution patterns of metabolites to the differences between control and tumor samples

Univariate and multivariate statistical analyses of metabolite levels were employed to assess the discrimination accuracy between tumor and the paired adjacent normal tissue. These analyses also enabled identification of significantly elevated levels of polar small molecules in kidney cancer (Supplementary material Table S3). Metabolite concentrations obtained by NMR following log-transformation and auto-scaling were analyzed with principal component analysis (PCA) to assess whether the patient versus control groups could be separated based on distinct metabolite profiles. Resulting 2D and 3D PCA scores plots (Fig. 2a, b, respectively) showed that the metabolite profiles of cancer patients are to a large extent dissimilar from controls, with PC1 and PC2 accounting for 39.9% and 9.1% of the variance, respectively. Only few outliers were detected in the central 95% of the field of view. One pair of tissue samples identified as outliers was removed.

Group separations were also inspected using OPLS-DA which revealed a clear separation between kidney tumors and the paired adjacent tissues. The classification from OPLS-DA score plot is shown in Fig. 2c. To evaluate the statistical robustness of this model 2000-permutation tests were conducted. Decent discrimination was observed between kidney cancer and normal tissues from the same patient (Q^2^ = 0.72, R^2^Y = 0.894, p-value < 5E − 04 (0/2000)), revealing substantial differences in metabolic profiles of cancer and normal renal tissues (Fig.S1, Supplementary material).

An OPLS-DA statistical method referred to as S-plot was employed to identify metabolites present in significantly different levels in kidney tumors and adjacent normal tissues. S-plot of kidney tumor tissues vs. control was shown in Fig. 2d. Higher values of |p(corr)| indicate metabolites that are more important to the classification. Variables with |p(corr)| value greater than 0.5 were considered significant. A paired t-test was performed for all variables. Five variables (listed in Table 1) were negatively correlated to group separation showing p(corr)[1] score of < − 0.5. Analysis of the S-score of the OPLS-DA model, combined with statistical paired t-test analysis (p values < 0.05) indicate these five metabolites responsible for differences (Fig. 2c; Table 1). OPLS-DA analysis revealed statistically lower levels of fumarate, leucine, sarcosine and phenylalanine in the cancer tissues compared to adjacent control tissues. Concentration data for the set of 48 metabolites with mean concentrations statistical parameters are reported in Table S3 (Supplementary material).

**Table 1:** Summary of the subsets of potential biomarkers of kidney cancer (p-value < 0.05; |p(corr)|> 0.5)

Comparison mode	Data set	Metabolite/element	*m/z*^a^	Adduct type	Mass error [ppm]	AUC	p(corr)	P-value^b^	Fold change^c^
Normal vs. Tumor	^1^H NMR	Fumarate^d^				0.91	− 0.681	4.1E − 11	3.1
		Leucine^d^				0.90	− 0.592	2.6E − 11	2.2
		Sarcosine^d^				0.89	− 0.617	7.7E − 09	3.8
		Tryptophan^d^				0.86	− 0.576	3.9E − 08	2.5
		Phenylalanine^d^				0.78	− 0.501	6.1E − 06	1.8
Benign vs. Malignant	^1^H NMR	Glucose^d^				0.89	0.609	2.96E − 05	0.2
		Creatine^d^				0.77	− 0.575	7.36E − 03	3.9
Normal vs. Tumor	ICP-OES	Zn^d^				0.94	− 0.866	7.99E − 06	2.4
		S^d^				0.84	− 0.750	2.59E − 05	1.2
		Na^d^				0.73	− 0.510	2.47E − 03	1.2
Normal vs. Tumor	^109^Ag LDI MS	Hydroxyeicosatrienoic acid^e^	361.208	[C_20_H_34_O_3_ + K]^+^	− 15.5	0.79	− 0.569	7.84E − 06	8.7
		UN^f^	121.122	–	–	0.78	− 0.586	1.34E − 05	42.3
		Octanediol^e^	147.137	[C_8_H_18_O_2_ + H]^+^	− 6.5	0.77	− 0.524	2.36E − 05	6.9
		Diethoxypentane^e^	161.153	[C_9_H_20_O_2_ + H]^+^	− 3.8	0.77	− 0.501	7.84E − 06	8.3
		UN^f^	243.824	–	–	0.77	0.518	1.03E − 05	0.8
		UN^f^	331.568	–	–	0.75	− 0.522	5.46E − 05	2.8
		UN^f^	120.940	–	–	0.76	− 0.516	9.39E − 06	1.7
		Oxoalanine^d^	141.991	[C_3_H_5_NO_3_ + K]^+^	6.3	0.84	− 0.568	8.95E − 07	4.0
		UN^f^	142.057	–	–	0.75	0.572	6.54E − 06	0.4
		1-(Methylthio)ethyl- -2-propenyl disulfide^e^	181.015	[C_6_H_12_S_3_ + H]^+^	− 13.2	0.75	0.569	2.35E − 06	0.3
		UN^f^	491.781	–	–	0.75	0.560	3.31E − 05	0.3

^a^Experimental monoisotopic mass; ^b^p-value determined from Student’s t-test, ^c^fold change between normal and tumor tissue calculated from the tissue weight-normalized concentration (NMR and ICP-OES data) or abundance (LDI MS data) mean values for each group; ^d^Identified compounds; ^e^Putatively annotated compounds; ^f^Unknown compounds

**Fig. 3:**
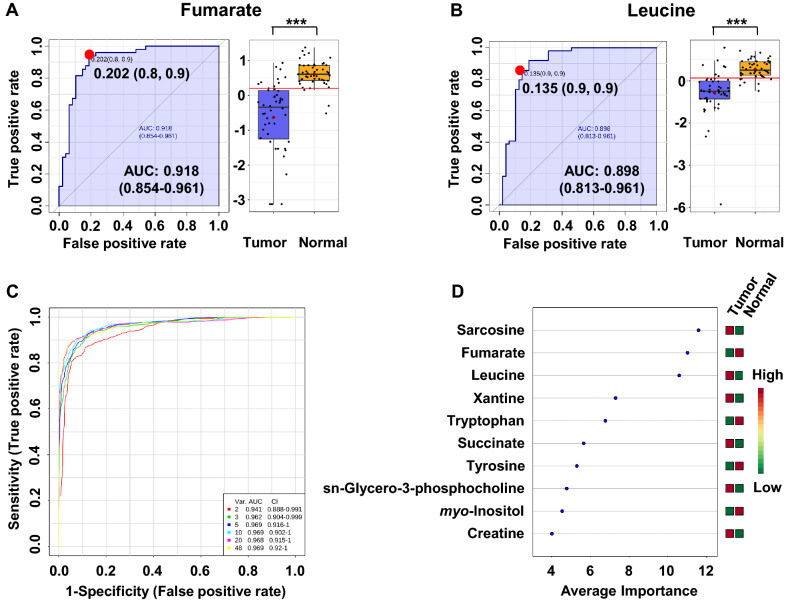
ROC curves for the predictive model based on ^1^H NMR data. **a**, **b** ROC curves of metabolites significantly associated with kidney cancer. **c** A combination of metabolites—model calculated with the use of logistic regression analysis. **d** Metabolites with the highest ability to discriminate tumor kidney tissue against controls

Next, univariate ROC curves were generated to characterize the predictive value of selected metabolites independently. The quality of the ranking represents the area under the curve (AUC). The results of univariate ROC curve analyses indicated that in the tissue samples, all five previously selected metabolites (fumarate, leucine, sarcosine, tryptophan and phenylalanine) exhibit high AUC above 0.78. Among these metabolites, the best ROC analyses with highest significance were achieved for fumarate (AUC = 0.918, specificity = 0.8 and a sensibility = 0.9), followed by leucine (AUC = 0.898, specificity = 0.9 and a sensibility = 0.9), sarcosine (AUC = 0.887, specificity = 0.9 and a sensibility = 0.9) and tryptophan (AUC = 0.857, specificity = 0.8 and a sensibility = 0.9) The range of concentrations for two first individual metabolites in the tissue samples of cancer patients compared to normal controls is shown in Fig. 3a and b.

A multivariable ROC analysis was performed to further assess the predictive value of these metabolites as a group to discriminate between tumor and normal tissues of each patient. The classification model was built using the MetaboAnalyst software and it’s associated the random forest algorithm. Figure 3c demonstrated that using a combination of metabolite level changes was a better discriminator (AUC > 0.94) than comparing each metabolite level change separately. An excellent discriminating classification was obtained when the three metabolites sarcosine, fumarate and leucine (AUC of 0.962) were considered all together, with a confidence interval (CI) from 0.904 to 0.999 (Fig. 3d). ROC curve illustrating the performance of the NMR models in distinguishing between tumor and normal tissue using random forest algorithm on five chosen potential metabolite biomarkers is shown in Fig. S2 (Supplementary material). AUC of these five compounds was 0.969 demonstrating high specificity and sensitivity to distinguish the groups. The permutation test with predictive accuracy as performance measure and 1000 permutations showed a p value < 0.001. The average of predicted class probabilities of each sample and the average accuracy from predictive accuracy test of the ROC curve show a good classification of samples. Most of the samples were classified in their respective group (Fig. S2, Supplementary material). These results suggest that assessing the levels of these three specific metabolites in kidney tissue could significantly increase the diagnostic performance of this model and could be used as diagnostic biomarkers to distinguish with high specificity and sensitivity, cancerous from healthy tissue in patients diagnosed with kidney cancer.

### Distinguishing between type of kidney cancer with ^1^H NMR

**Fig. 4:**
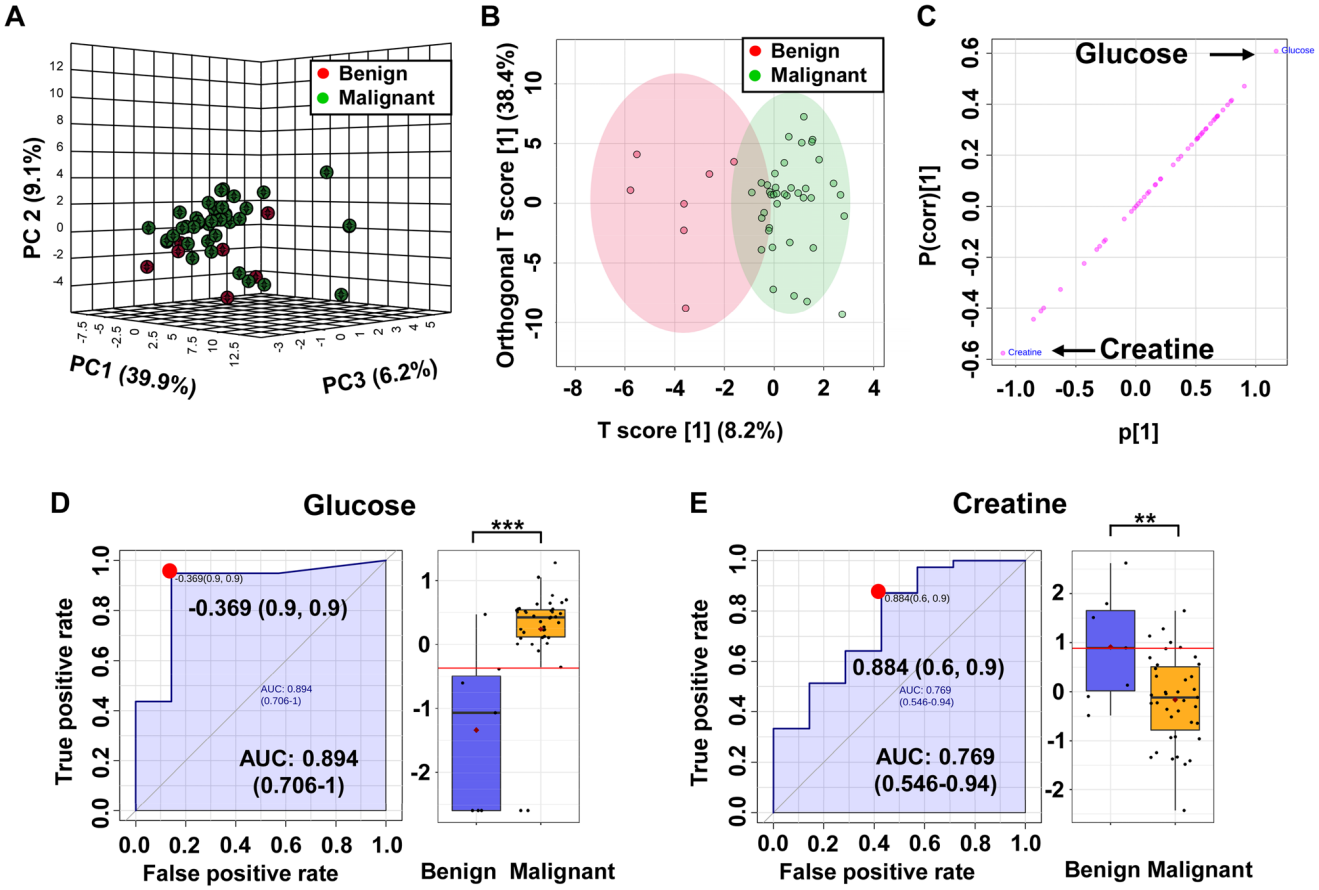
Discrimination between benign and malignant kidney cancer based on the metabolic profiles of kidney tissue analyzed by ^1^H NMR spectroscopy. **a** 3D PCA and **b** OPLS-DA scores plots generated from the NMR data of the benign (red) and malignant (green) tumors. **c** The OPLS-DA loading S-plot showing the distribution patterns of metabolites to the differences between benign and malignant samples; **d**, **e** ROC curves of metabolites significantly associated with differentiation between types of kidney cancer

^1^H NMR metabolomics analysis of tissue samples was employed to evaluate whether distinct metabolic trends allow to distinguish between types (benign and malignant) of kidney cancers. A 3D PCA scores plot revealing a good discrimination between benign and malignant cases is shown in Fig. 4a.

Comparing benign versus malignant tissue with OPLS-DA revealed a very good discrimination between these two groups (Fig. 4b). Quality factors for this model were: Q^2^: 0.395 and R^2^Y: 0.701 with p values based on permutation tests lower than 0.05. Detailed results are shown in supplementary material Fig.S3 (Supplementary materials). Analysis of S-plots revealed that glucose and creatine are significant contributors to the separation between malignant vs benign samples (Fig. 4c, Table 2). In this study glucose was positively correlated with group separation with a p(corr)[1] score > 0.5 and creatine negatively correlated with a p(corr)[1] score < − 0.5. Biomarker candidates were further subjected to t-test analysis to assess the significance of altered levels of these metabolites in benign versus malignant tissue samples. In total, 2 of the identified metabolites were found at statistically significant differential concentrations (p < 0.05; q < 0.05 and |p(corr)|> 0.5), suggesting that examining the differential levels of glucose and creatine may be an effective way to identify malignant tissue and discriminate from healthy tissue within tissue samples of kidney cancer patients. Univariable ROC curve analyses were conducted to further assess the predictive value of these metabolite level changes. AUC values for glucose were found to be as high as 0.894 (Fig. 4d) and 0.769 for creatine (Fig. 4c). Additionally, ROC curve for only these two selected metabolites were constructed with an AUC value of 0.889 (Fig. S4, Supplementary material). The permutation based on measure area under ROC curve test showed a p value = 0.003. The average of predicted class probabilities of each sample across the 100 cross-validations indicates that most of the samples were classified in their respective group. The average accuracy based on 100 cross validations was 0.848. These results support that glucose and creatine may be good discriminating indicators of kidney tumor types.

### Elemental profile of tissue in kidney cancer with ICP-OES

**Fig. 5:**
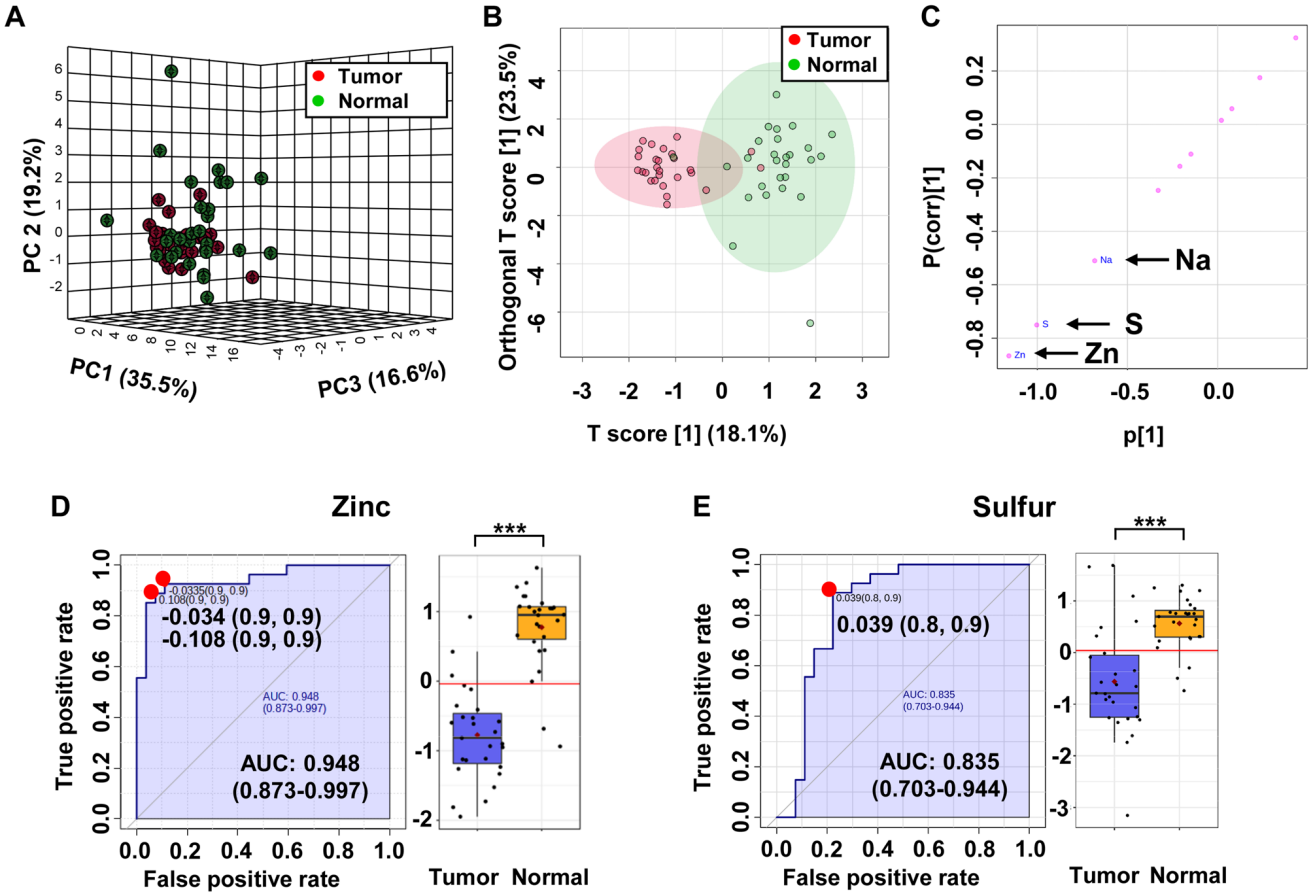
Tissue metallomic profiles based on ICP-OES. **a** 3D PCA and **b** OPLS-DA scores plots generated from the ICP-OES data of the tumor tissue (red) and adjacent control tissue (green) samples. **c** The OPLS-DA loading S-plot showing the distribution patterns of elements to the differences between tumor and control tissue samples; **d**, **e** ROC curves of chosen elements significantly associated with kidney cancer

Chemical elements concentrations obtained from ICP-OES experiments on 58 extracts of kidney tissue samples (28 cancer and 28 control tissues) were subjected to statistical data analysis. Elemental mean concentrations in tumorous and non-tumorous tissues are summarized in Table S4 (Supplementary material) for each element. As shown in Fig. 5a, the 3D-PCA scores plot reveals a separation trend between the two groups. Results from OPLS-DA analysis, shown in Fig. 5b, provide a much clearer separation (compared to the PCA analysis) between tumor and normal tissues with high explanative and predictive parameters of this model: R^2^X and Q^2^ were 0.753 and 0.649 respectively (Fig.S5, Supplementary material).

Elements of interest were selected from the S-plot profile, constructed from the OPLS-DA model (Fig. 5c). The loading S-plot revealed that three variables were negatively correlated with group separation showing -p(corr)[1] < − 0.5. According to p-values and p(corr) scores, three discriminate variables (Na, S and Zn) were found to be potentially good discriminators of in kidney tissues (Table 1). ROC analysis revealed that zinc exhibited the highest significance with an AUC value of 0.948, sensitivity of 0.9, and specificity of 0.9 (Fig. 5d), and sulfur exhibited an AUC value of 0.835, sensitivity of 0.8, and specificity of 0.9 (Fig. 5e). Furthermore, ROC curve plot illustrating the performance of the ICP OES model in distinguishing between tumor and normal tissue with only three selected elements (S, Zn, Na) was performed. AUC value was 0.953 which indicates very good discriminatory ability (Fig. S6, Supplementary material). The permutation based on area under ROC curve test showed a p value < 0.001. The average of predicted class probabilities of each sample across the 100 cross-validations indicates that most of the samples were classified in their respective group. The average accuracy based on 100 cross validations was 0.899. Most of the samples were classified in their respective group (Fig.S5, supplementary material). Levels of sulfur and sodium were identified as potentially good indicators of kidney cancer, while zinc concentration could be considered an excellent kidney cancer biomarker.

### Metabolic profiling of kidney cancer tissue with ^109^AgNPET LDI MS

Laser mass spectrometry-based approach was also applied to investigate the tissue metabolic profiles of patients with kidney cancer. Statistical analysis was performed separately for polar and non-polar metabolite extracts of kidney tissue. 575 and 461 common features were detected in the non-polar and polar extracts of tissues samples, respectively of 50 patients with kidney cancer by ^109^AgNPET LDI mass spectral analyses. Spectral intensity data from LDI MS spectra was subjected to multivariate data analysis to assess whether these could discriminate between tissue types. 2D-PCA and OPLS-DA score plots were generated for the entire data sets, and 2D-PCA scores plots of mass spectral features revealed moderate discrimination between tumor and normal tissue (Fig.S7, supplementary material). Results from OPLS-DA analysis, shown in Fig.S7B, D (supplementary material), presented a clear separation between two groups suggesting that the ^109^AgNPET LDI MS-based tissue metabolomics model can also be used to effectively identify discriminating metabolic differences that separate kidney tumor and normal tissues. All relevant spectral features and observations resulting from the ^109^AgNPET LDI MS-based metabolomics analyses are reported in Table 1.

Potential features for group separation of non-polar and polar extracts of tissue samples were subsequently identified by S-plot loading analysis of corresponding OPLS-DA models, and based on significance criterion of |p(corr)|> 0.5 and |p|> 0.05 (Fig.S8 A, D, Supplementary material). The S-plot loading for the non-polar extract analysis revealed one variable as positively correlated with group separation, exhibiting a + p(corr)[1] value > 0.5 and five variables as negatively correlated with group separation, and exhibiting a—p(corr)[1] value < − 0.5. Resulting R^2^Y and Q^2^ parameters assessing the validity of the OPLS-DA model consisted of 0.905 (p-value < 5E − 04 (0/2000)) and 0.337 (p-value < 5E-04), respectively (Fig. S8 B, Supplementary material). Mean abundances of spectral features identified in the non-polar extracts of tissue samples are reported in Table S5 (Supplementary material). The S-plot loading for the polar extracts revealed three variables as positively correlated with group separation and + p(corr)[1] > 0.5, and two negatively correlated with group separation and—p(corr)[1] < -0.5. Resulting R^2^Y and Q^2^ values were respectively 0.905 (p-value < 5E − 04 (0/2000)) and 0.337 (p-value < 5E − 04), providing a robust indication of goodness of fit and predictability of the two-class OPLSDA model (Fig.S8 E, Supplementary material). Mean abundances of *m/z* variables identified to be significantly different in the non-polar extracts of tumor vs normal tissue samples are reported in Table S6 (Supplementary material).

Selected *m/z* values were subjected to multivariate ROC curve analyses based on random forest algorithms. All eleven previously selected *m/z* mass spectral features were identified to exhibit high AUC values with an area under the curve > 0.75 (Fig.S9, Supplementary information). This analysis was followed by a multivariate ROC analysis to assess whether groups of spectral features were better predictors compared to when features were analyzed separately. As shown in Figures S10 (Supplementary material), the combination of mass features in non-polar extracts was found to be a better discriminator between healthy and malignant tissue than the individual analysis of each mass feature. A good classification was obtained with ten features (AUC of 0.82), with confidence intervals (CI) ranging from 0.729 to 0.906. A good classification was also obtained for spectral features from polar extracts of kidney tissue, with ten of these features exhibiting AUC values of 0.818 and CI ranging from 0.705 to 0.911. For both MS models features with the highest ability of contributions to classification accuracy are shown in Fig.S10 B and D (Supplementary material). These results suggest that selected mass features can significantly increase the diagnostic performance of MS model and could be used as diagnostic biomarkers that separate cancer tissue samples from normal with high specificity and sensitivity.

ROC curves illustrating the performance of the ^109^AgNPET LDI MS model in distinguishing between tumor and normal tissue using random forest algorithm on six selected features from non-polar and five features from polar extracts of studied tissues were presented in figures S11 and S12 (Supplementary material). Putative identifications were guided by searches on various metabolite databases i.a. HMDB (Wishart et al., 2007), MetaCyc (Caspi et al., 2018), LipidMaps (Sud et al., 2007), and Metlin (Smith et al., 2005). Five tissue mass features were putatively identified as known metabolites. All important mass spectral features and metabolite ID resulting from the ^109^Ag LDI MS analyses are reported in Table 1.

### Distinguishing between age and gender

To evaluate the significant changes between tissue extracts from patients of different sex and age the analysis of variance (ANOVA) was performed. The analysis of gender and age differentiation was performed for data obtained from all three analytical platforms. The analysis was performed using samples in four groups; for gender discrimination samples from female, female control, male and male control were used. For age discrimination samples from patients of age under 60 and over 60 were used. Data was presented in supplementary materials (Figures S13–S18). However, in all cases, no statistically significant differences were observed between patients of different sex and age. It has been observed that data from tumor tissue extracts within each group (male vs female and age > 60 *vs* age < 60) differed only compared to the adjacent extracts of control normal tissues. This is mainly due to the insufficient number of patients under 60 and of the female sex because the population studied was relatively old and mainly male.

### Pathway analysis of potential biomarkers

**Fig. 6:**
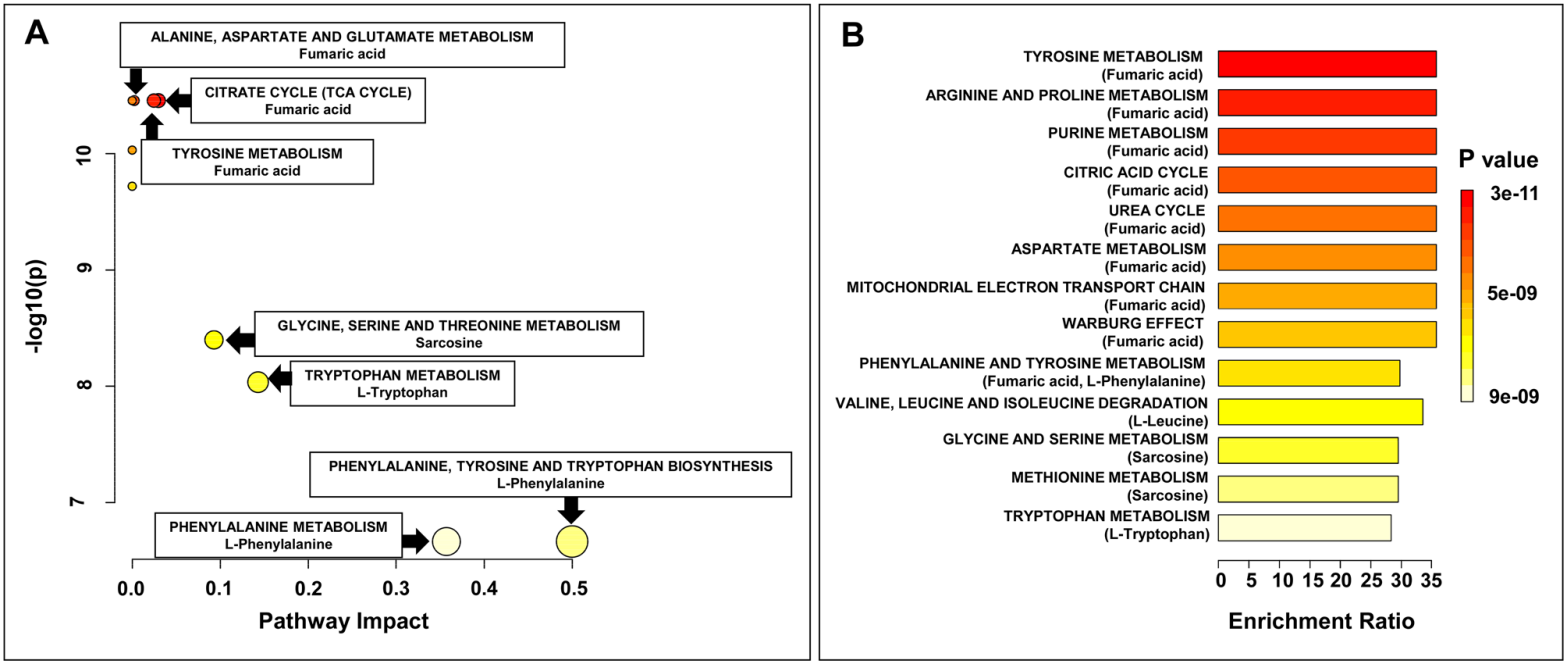
Results of pathway topology analysis of selected five differential metabolites statistically significant in RCC (found in NMR spectra). **a** Pathway analysis based on KEGG; bubble area donating to the impact of each pathway with color representing the significance from highest in red to lowest in white; **b** Quantitative enrichment analysis based on SMPDB

The most differentiating metabolites between kidney cancer and normal tissues among three analytical platforms were subjected to pathway analysis by MetaboAnalyst 5.0. However, only the compounds selected in the NMR analysis turned out to be important in the metabolic pathways in the human body, therefore the quantitative data from this platform was used to identify the most relevant pathways involved in the kidney cancer. The concentrations of fumarate, sarcosine, leucine, tryptophan and phenylalanine were subjected to pathway analysis and quantitative enrichment analysis using a MetaboAnalyst 5.0. 12 metabolic pathways, including tyrosine metabolism, arginine and proline metabolism, purine metabolism, citric acid cycle, urea cycle, aspartate metabolism, mitochondrial electron transport chain, Warburg effect, phenylalanine and tyrosine metabolism, valine, leucine and isoleucine degradation, glycine and serine metabolism, methionine metabolism and tryptophan metabolism were significantly related to kidney cancer. The result of pathway analysis is shown in Fig. 6a and Table S7 in Supplementary materials. Furthermore, in order to expand metabolomic analysis of pathway related to kidney cancer, the quantitative enrichment analysis module in MetaboAnalyst, with extensive list of pathways from SMPDB database was performed. Concentrations of 5 metabolites, identified from the global metabolomic profiling, were entered as input data. It was found that 6 additional pathways including aspartate metabolism, methionine metabolism, mitochondrial electron transport chain, purine metabolism, urea cycle and Warburg effect significantly related to kidney cancer (Fig. 6b; Table S8, Supplementary materials). The power of metabolic pathways analysis was confirmed by p-value Holm p-values and FDR of less than 0.001 for every pathway (Tables S7 and S8, Supplementary material).

## Discussion

In this study, ^1^H NMR, ICP-OES and ^109^Ag LDI MS were employed to assess similarities and differences in metabolite and selected chemical elements abundances between renal tumors and normal parenchymal tissue. ^1^H NMR metabolomics analysis revealed significant differences in the concentration of essential amino acid, including leucine, tryptophan and phenylalanine. Essential amino acids cannot be synthetized de novo by the human cells thus must be provided by diet and intestinal microbiota. Rapid and uncontrolled proliferation of cancer requires increased levels of amino acids to be used as basic building blocks for the synthesis of new proteins. In this study, depletion of essential amino acids is reflected in decreased concentration of leucine, tryptophan and phenylalanine in cancer tissue. Our results are consistent with the findings of Jing et. al. who also showed decreased level of numerous amino acids in RCC tissue specimens (Jing et al., n.d.).

Decreased levels of tryptophan may also be associated with its excessive consumption. Tryptophan metabolism plays a major role in cancer resistance to immunotherapeutic treatment, as it is metabolized to kynurenine via the kynurenine pathway. Kynurenine activates the transcription factor aryl hydrocarbon receptor (AhR) which induces immunosuppression by disrupting the ability of dendritic cells and T cells to eliminate cancer cells. This may also partially explain the high rate of IFNα therapy failure in RCC (Trott et al., 2016). Another amino acid derivative whose concentration was lower in RCC specimens is sarcosine, which can be converted to glycine by action of sarcosine dehydrogenase. It has been reported that elevated levels of sarcosine can be detected in the urine of the patients with prostate cancer (Sreekumar et al., 2009). However further studies failed to prove its value in prostate cancer diagnosis (Jentzmik et al., 2010).

ICP-OES analysis was conducted to assess potential differences in chemical elements abundance between cancer and control tissues. ICP-OES measurements revealed decreased concentrations of Zn, S and Na in the tissue of renal tumors. Numerous elements have been implicated in the process of tumorigenesis, as heavy metals like arsenic, cadmium, chromium, and nickel are considered carcinogenic in humans (Kim et al., 2015). On the other hand, metals like zinc (Zn) and sodium (Na) are considered essential for normal human body functions. Zinc is the second most abundant metal in the human body, and is essential for over 300 enzyme functions including carbonic anhydrase, superoxide dismutase and alkaline phosphatase. Moreover, zinc is important in DNA and RNA metabolism, signal transduction, gene expression and protein folding via action of zinc finger motifs. This study demonstrated a decreased concentration of zinc in kidney tumors when compared to normal renal parenchyma. Moreover, zinc showed highest predictive value of all detected elements/metabolites with AUC of 0.948. Two studies evaluating the zinc level in the kidney tissue reported decreased levels of zinc in cancer samples. However, this relation was found to be statistically non-significant due to the small group size of the studies (Dobrowolski et al., 2002). Studies by Abdel-Gawad, Reddy and Calvo reported a statistically significant increase in zinc concentration in RCC specimen (Abdel-Gawad et al., 2020; Calvo et al., 2009; Reddy et al., 2003). On the other hand, studies by Dobrowolski et al. which evaluated zinc levels in kidney tissue reported a decreased level of zinc in cancer samples (Dobrowolski et al., 2002), providing cofounding results as to zinc level trends in cancer tissue.

Association between high concentration of heavy metals in cancer tissue and cancerogenesis have been extensively studied. Most studies agree that exposure to certain heavy metals, particularly arsenic, nickel, cadmium, chromium may increase the risk of cancer. Unfortunately, the causative mechanism is still unknown. Some studies hypothesize that heavy metals may induce cellular hypoxia through activation of hypoxia inducible factor 1 (Galanis et al., 2009; Osipyants et al., 2018). This may be important concerning pharmacological treatment of renal cancer which is based on the inhibition of HIF-1 induced overexpression of the products of tyrosine kinase incl. VEGF and PDGF. First line treatment of metastatic RCC is based on targeted therapy against tyrosine kinase and include tyrosine kinase inhibitors (sunitynib, pazopanib, axitinib, cabozantinib, lenvatinib and tivozanib) and monoclonal antibodies targeting VEGF (bevacizumab) (Ljungberg et al., 2007) Therefore, it may be hypothesized that chelation of heavy metals which may act as HIF-1 activators could improve efficacy of the treatment. Another important question is why elevated concentration of heavy metals in cancer tissue is observed. Romaniuk et al. suggested that this may be due to an impaired excretion of metabolites and heavy metals by cancer cells (Romaniuk et al., 2017). However, in this study, we observed decreased concentration of metals like sodium and zinc in cancer tissue. Sodium is the primary cation in the extracellular fluid and its main role is in regulation of water homeostasis between cells and extracellular space. This is made possible through a variety of sodium channels that operate between extra and intracellular space. This observed decrease in concentration of sodium concentration may be caused by dysfunction of those channels. Decreased concentration of zinc in cancer tissue was also observed by Dobrowolski et al. (2002) (Kwiatek et al., 1996) Studies have reported that zinc exerts a cytotoxic effect on cancer cells (Costello & Franklin, 2005). Therefore, the decreased concentration of zinc in cancer tissue may be explained by an attempt to avoid cytotoxic effects on the malignant cells. Diagnosis of renal cell carcinoma subtype is based on histopathological evaluation of resected/biopsied tissue. Heavy metals are ubiquitous in the human body and therefore are not specific for cancer tissue and therefore they cannot be utilized alone as cancer biomarkers. However, in combination with other metabolites they may provide unique information concerning prognosis and staging.

Sodium is the primary cation in the extracellular fluid (EF), which includes interstitial fluid—i.e. fluid surrounding cells and intravascular fluid (blood plasma). Blood plasma sodium level varies between 135 and 145 mmol/L, whereas intracellular levels are maintained at 12 mmol/L. Sodium’s major role is the regulation of water homeostasis in the human body, and is also essential for mediating electrical signaling of neuronal cells. However, in solid tumors, like kidney cancer, impaired growth of new blood vessels leading to hypoxia and themetabolic reprogramming of cancer cells favoring aerobic glycolysis (Warburg effect) leads to the development of an intracellular acidic environment. To maintain intracellular pH, excess H^+^ must be secreted outside the cell or neutralized by importing bicarbonate (HCO_3_^−^). These processes are mediated by Na^+^/H^+^ antiport. systems and Na^+^, HCO_3_^−^ co-transporters which are coupled to intracellular Na^+^ transport (Stock & Pedersen, 2017). In this study we observed decreased concentration of sodium within kidney tumor tissue, reflecting changes in sodium levels in both extracellular and intracellular space. However, the intracellular volume fraction accounts for 80% of total tissue volume (Madelin & Regatte, 2013). Therefore, this concentration decrease reflects mainly a decrease of sodium in intracellular space, which could be due to impairment of sodium channel function in cancer cells.

^109^Ag LDI MS analysis revealed significance differences in abundance of 12 metabolites between kidney tumors and control. Six of them were putatively identified as: hydroxyeicosatetraenoic acid, octanediol, diethoxypentane, oxoalanine, 1-(methylthio)ethyl-2-propenyl disulfide, hydroxyeicosatetraenoic acid, octanediol, diethoxypentane and oxoalanine were found to be in higher concentration in cancer tissue whereas 1-(methylthio)ethyl-2-propenyl disulfide was higher in normal renal tissue.

Hydroxyeicosatetraenoic acid is an eicosanoid that is produced by enzymatic oxidation of arachidonic acid. It has been reported that 20-hydroxyeicosatetraenoic acid (20-HETE) is associated with cancerogenesis. Moreover, selective inhibitors of the 20-HETE-producing enzymes (CYP4A and CYP4F) can inhibit growth of numerous cell lines including renal cell carcinoma (Alexanian & Sorokin, 2013). These findings are consistent with our observations that renal tumors contain higher levels of hydroxyeicosatetraenoic acid compared to normal renal tissue.

In this study glucose and creatine concentration were shown to discriminate between benign and malignant kidney tumors, with higher glucose concentrations found in cancerous tissue. Numerous studies have reported the accumulation of glucose in RCC tumors as a result of the metabolic reprogramming of RCC cells. It is believed that this is due to enhanced uptake of glucose resulting from overexpression of GLUT-1 glucose transporters under hypoxic environment (Lucarelli et al., 2015; Nakaigawa et al., 2017; Popławski et al., 2017). This hypothesis is supported by Chan et al. who showed that inhibition of GLUT-1 transporters starves RCC cells by depleting glucose supply (Chan et al., 2011). These observations also implicate that benign kidney tumors are more metabolically similar to normal tissue than malignant tumors.

In contrast to glucose, creatine was found in lower concentration in cancerous tissue. Creatine, a non-protein-derived amino acid, is crucial for energy storage as phosphocreatine is used to regenerate ATP. Study conducted on mice by Biase et al. showed that creatine uptake is important for the anti-tumor activities of CD8 T-cells (Di Biase et al., 2019). Lower concentration of creatine in cancer tissue may impair immune functions that are essential to fight cancer. In this study, a metabolite that the most discriminated between tumor and control tissue was fumarate. It was found in lower concentration in tumor tissue. The analysis of the biochemical pathways indicates that fumarate participates in ten metabolic pathways including alanine, aspartate and glutamate metabolism, arginine and proline metabolism, aspartate metabolism, citrate cycle, mitochondrial electron transport chain, purine metabolism, pyruvate metabolism, tyrosine metabolism, urea cycle and is also important in Warburg effect. Another metabolite, sarcosine was significant in pathways such as glycine, serine and threonine metabolism and methionine metabolism. Leucine participates in significantly changed KEGG and SMPDB pathways of aminoacyl-tRNA biosynthesis, valine, leucine and isoleucine biosynthesis and degradation. Significant KEGG and SMPDB pathway including aminoacyl-tRNA biosynthesis, phenylalanine, tyrosine as well as tryptophan biosynthesis are related to phenylalanine.

## Conclusion

This work has demonstrated that value of high-resolution ^1^H NMR, ICP-OES and ^109^AgNPET LDI MS, along with multivariate statistics to characterize kidney tissue metabolome and metallome differences between tumor and normal tissue of patients suffering from kidney cancer. With regard to biomarker discovery, five potentially robust metabolic biomarkers in 49 tumor tissue samples of kidney cancer patients and 49 adjacent normal tissues treated as controls were identified using ^1^H NMR spectroscopy, while 11 mass spectral features were identified from nanoparticle-based LDI mass spectrometry analyses. The most important endogenous compounds and trace elements having bioactive properties and pharmacological applicability were discussed in details. Moreover, we also demonstrated the possibility of discriminating between different kidney cancer types using ^1^H NMR metabolomics. This study also supports the value of integrated NMR and mass spectrometry to identify candidate biomarkers and characteristic changes in small molecule metabolite levels which could prove to be very valuable for use as diagnostics or to track disease progression, offering less invasive ways to screen patients with kidney cancer.

## Supplementary Information

Below is the link to the electronic supplementary material.

Supplementary file1 (DOCX 5903 KB)

## Data Availability

The data that support the findings of this study is available from the corresponding author upon reasonable request.

## Declarations

### Research involving human and/or animal participants

This article does not contain any studies with human and/or animal participants performed by either of the authors.
