# Supplementary material for: Metabolomic and elemental profiling of human tissue in kidney cancer
Source: Metabolomics. 2021 Mar 4;17(3):30. doi: 10.1007/s11306-021-01779-2 (PMC7932981; doi:10.1007/s11306-021-01779-2)
Supplement: Supplementary file 1 — Supplementary file1 (DOCX 5903 KB) [file 11306_2021_1779_MOESM1_ESM.docx]

Supplementary material

**Metabolomic and metallomic profiling of human tissue in kidney cancer**

Joanna Nizioł^a*^, Valérie Copié^b^, Brian P. Tripet^b^, Leonardo B. Nogueira^c^, Katiane O.P.C. Nogueira^d^, , Krzysztof Ossoliński^e^, Adrian Arendowski^a^ and Tomasz Ruman^a^

*^a^Rzeszów University of Technology, Faculty of Chemistry, 6 Powstańców Warszawy Ave., 35-959 Rzeszów, Poland, e-mail: jniziol@prz.edu.pl*

*^b^The Department of Chemistry and Biochemistry, Montana State University, Bozeman, Montana 59717, United States*

*^c^Federal University of Ouro Preto, Department of Geology, Ouro Preto, Minas Gerais, Brazil*

*^d^Federal University of Ouro Preto, Department of Biological Sciences, Ouro Preto, Minas Gerais, Brazil*

*^e^Department of Urology, John Paul II Hospital, Grunwaldzka 4 St., 36-100 Kolbuszowa, Poland*

*Corresponding author: Joanna Nizioł, e-mail: jniziol@prz.edu.pl, tel: (+48 17) 865-1896

**Table of contents**

**Table S1.** Clinical characteristic of kidney cancer patients **3**

**S1.** NMR Spectra Acquisition and Preprocessing**4**

**S2.** NMR Data Analysis**5**

**Table S2.** ICP-OES parameters, conditions of analysis and results for reference material **6**

**Figure S1.** Results of permutation test of OPLS-DA for ^1^H NMR data set **6**

**Figure S2.** ROC curve analysis of the NMR model in distinguishing between tumor and normal tissue on five chosen potential metabolite biomarkers**7**

**Table S3.** Mean metabolite concentrations for normal *vs*. cancer kidney tissue extracts on ^1^H NMR data set **8**

**Figure S3.** Results of permutation test of OPLS-DA for ^1^H NMR data set from different types of kidney cancer **10**

**Figure S4.** ROC curve analysis of the NMR model in distinguishing between benign and malignant tumor on two chosen potential metabolite biomarkers **12**

**Table S4.** Mean metabolite concentrations for normal *vs*. cancer kidney tissue extracts on ICP-OES data set **10**

**Figure S5.** OPLS-DA model validation of ICP-OES data set **13**

**Figure S6.** ROC curve analysis of the ICP OES model in distinguishing between benign and malignant tumor on three chosen elements **14**

**Figure S7.** Tissue metabolite profiles for non-polar and polar extracts of tissue kidney cancer group based on ^109^Ag NPET LDI MS. **15**

**Figure S8.** OPLS-DA details of the ^109^AgNPET LDI MS data from non-polar and polar extracts of kidney cancer tissue. **16**

**Table S5.** Mean metabolite abundance for normal *vs*. cancer kidney tissue non-polar extracts based on LDI MS data set.**17**

**Table S6.** Mean metabolite abundance for normal *vs*. cancer kidney tissue polar extracts based on LDI MS data set.**19**

**Figure S9.** ROC curve analysis for potential biomarkers predicted by classical univariate analysis of data from ^109^AgNPET LDI MS for non-polar and polar extracts of studied tissues **20**

**Figure S10.** ROC curve analysis for all non-polar and polar extracts of tissue kidney cancer group based on ^109^Ag NPET LDI MS**21**

**Figure S11.** ROC curve analysis of the ^109^AgNPET LDI MS model in distinguishing between tumor and normal tissue using random forest algorithm on five selected features from polar extracts of studied tissues **22**

**Figure S12.** ROC curve analysis of the ^109^AgNPET LDI MS model in distinguishing between tumor and normal tissue using random forest algorithm on five selected features from non-polar extracts of studied tissues **23**

**Figure S13.** Metabolite profiles based on ^1^H NMR spectra of tissue extracts from male and female patients with kidney cancer **24**

**Figure S14.** Metabolite profiles based on ^1^H NMR spectra of tissue extracts of patients under age 60 and over age 60 with kidney cancer **25**

**Figure S15.** Elemental profiles based on ICP-OES data of tissue extracts from male and female patients with kidney cancer**26**

**Figure S16.** Elemental profiles based on ICP-OES data of tissue extracts of patients under age 60 and over age 60 with kidney cancer **27**

**Figure S17.** Metabolite profiles based on ^109^AgNPET LDI MS data of tissue extracts from male and female patients with kidney cancer **28**

**Figure S18.** Metabolite profiles based on ^1^H NMR spectra of tissue extracts of patients under age 60 and over age 60 with kidney cancer **29**

**Table S7.** Result from Pathway Analysis **30**

**Table S8.** Result from Enrichment Pathway Analysis **31**

Table S1. Clinical characteristics of kidney cancer patients

| **Patient no.** | **Histopathological diagnosis of kidney cancer** | **Size** | | **Sex** | | **Age** | | **Staging** | | **Grading** | |
| --- | --- | --- | --- | --- | --- | --- | --- | --- | --- | --- | --- |
| 1 | ccRCC | 74x66x71 | | M | | 61 | | pT1aN0M0 | | G4 | |
| 2 | ccRCC | 63x42x60 | | M | | 89 | | pT4N0M1  (M:adrenal gland) | | G3 | |
| 3 | ccRCC | 118x89x105 | M | | 74 | | pT3aN0M0,  invades perirenal fat, invades renal vein | | G3 | |  |
| 4 | ccRCC | 51x54x82 | | M | | 73 | | pT3aN0M1  (M:adrenal gland, spinal cord), invades perirenal fat, invades renal vein | | G4 | |
| 5 | ccRCC | 74x61x72 | | M | | 72 | | pT1bN0M0 | | G2 | |
| 6 | pRCC | 68x60x60 | | M | | 78 | | pT1bN0M0 | | G2 | |
| 7 | meta – lung adenocarcinoma | 70x80x50 | | M | | 70 | | - | | - | |
| 8 | - | 31x25x30 | | M | | 44 | | - | | - | |
| 9 | ccRCC | 118x82x130 | | F | | 57 | | pT3aN0M1  (M:liver, lung), invades renal vein | | G2 | |
| 10 | chRCC | 55x72x61 | | M | | 73 | | pT2aN0M0 | | G3 | |
| 11 | ccRCC | 41x30x39 | | F | | 87 | | pT3aN0M0 | | G3 | |
| 12 | ccRCC | 55x51x57 | | M | | 77 | | pT3aN0M0,  invades perirenal fat | | G3 | |
| 13 | ccRCC | 23x26x20 | | F | | 69 | | pT1aN0M0 | | G2 | |
| 14 | pRCC | 17x14x15 | | M | | 75 | | pT1aN0M0 | | G3 | |
| 15 | AML | 23x24x32 | | M | | 61 | | pT1aN0M0 | | - | |
| 16 | ccRCC | 22x20x20 | | M | | 72 | | pT1aN0M0 | | G1 | |
| 17 | ccRCC | 22x19x25 | | F | | 77 | | pT1aN0M0 | | G2 | |
| 18 | oncocytoma | 25x23x24 | | F | | 70 | | pT1aN0M0 | | - | |
| 19 | oncocytoma | 27x22x21 | | M | | 56 | | pT1aN0M0 | | - | |
| 20 | ccRCC | 115x100x135 | | F | | 86 | | pT3aN0M0,  invades renal vein | | G3 | |
| 21 | ccRCC | 20x18x20 | | M | | 36 | | pT1aN0M0 | | G2 | |
| 22 | ccRCC | 25x20x20 | | M | | 52 | | pT1aN0M0 | | G2 | |
| 23 | ccRCC | 26x30x31 | | F | | 73 | | pT1aN0M0 | | G2 | |
| 24 | CDC | 25x25x38 | | M | | 70 | | pT1aN0M1 | | G3 | |
| 25 | oncocytoma | 28x28x30 | | M | | 74 | | pT1aN0M0 | | - | |
| 26 | r1:TCRC,  r2:AML | R1:25x18x20 R2:27x20x20 | | F | | 80 | | R1: pT1aN0M0,  R2: pT1aN0M0 | | - | |
| 27 | ccRCC | 30x20x15 | | M | | 57 | | pT1aN0M0 | | G1 | |
| 28 | ccRCC | 28x24x20 | | F | | 81 | | pT1aN0M0 | | G2 | |
| 29 | ccRCC | 38x33x32 | | F | | 65 | | pT1aN0M0 | | G2 | |
| 30 | ccRCC | 18x17x20 | | F | | 66 | | pT1aN0M0 | | G1 | |
| 31 | AML | 95x58x73 | | F | | 53 | | pT2bN0M0 | | - | |
| 32 | ccRCC | 20x20x20 | | M | | 66 | | pT1aN0M0 | | G2 | |
| 33 | ccRCC | 46x49x57 | | F | | 69 | | pT3aN1M1,  invades renal sinus fat | | G3 | |
| 34 | oncocytoma | 32x32x31 | | M | | 52 | | pT1aN0M0 | | - | |
| 35 | ccRCC | 62x76x80 | | M | | 80 | | pT2aN0M0 | | G3 | |
| 36 | ccRCC | 50x34x40 | | M | | 70 | | pT1bN0M0 | | G2 | |
| 37 | ccRCC | 66x48x54 | | M | | 73 | | pT3aN0M0,  invades renal vein | | G2 | |
| 38 | ccRCC | 72x80x83 | | M | | 76 | | pT3aN0M0,  invades renal vein | | G3 | |
| 39 | ccRCC | 20x20x20 | | M | | 74 | | pT1aN0M0 | | G3 | |
| 40 | SRC | 54x50x40 | | F | | 78 | | - | | - | |
| 41 | ccRCC | 21x20x20 | | M | | 63 | | pT1aN0M0 | | G2 | |
| 42 | ccRCC | 61x71x60 | | F | | - | | pT1bN0M0 | | G1 | |
| 43 | ccRCC | 64x49x50 | | F | | 78 | | pT3aN0M0,  invades renal vein | | G3 | |
| 44 | ccRCC | 23x19x20 | | M | | 51 | | pT1aN0M0 | | G2 | |
| 45 | ccRCC | 35x30x30 | | F | | 54 | | pT1aN0M0 | | G2 | |
| 46 | ccRCC | 40x36x40 | | F | | 81 | | pT1aN0M0 | | G1 | |
| 47 | chRCC | 25x20x20 | | M | | 85 | | pT1aN0M0 | | G1 | |
| 48 | ccRCC | 33x30x30 | | F | | 81 | | pT1aN0M0 | | G1 | |
| 49 | AML | 12x17x12 | | F | | 56 | | pT1aN0M0 | | - | |
| 50 | ccRCC | 37x32x35 | | M | | 68 | | pT1aN0M0 | | G2 | |

AML – angiomyolipoma; ccRCC - clear cell RCC; CDC - collecting duct carcinoma chRCC - chromophobe renal cell carcinoma; F – female; N0 - no nodal involvement; N1 - metastatic involvement of regional lymph node(s); M – male; M0 - no distant metastases; M1 - distant metastases; pRCC - papillary RCC; meta – metastasis; SRC - simple renal cyst; T1a - tumor confined to kidney, <4 cm; T1b - tumor confined to kidney, >4 cm but <7 cm; T2a - tumor confined to kidney, >7 cm but not >10 cm; T2b - tumor confined to kidney, >10 cm; T3a: Tumor grossly extends into the renal vein or its segmental branches, or tumor invades perirenal and/or renal sinus fat but not beyond the Gerota fascia; T4: involves ipsilateral adrenal gland or invades beyond Gerota's fascia; TCRC - tubulocystic renal cell carcinoma

**S1. NMR Spectra Acquisition and Preprocessing**

1D ^1^H NMR spectra were collected at 298 K (25°C) using a Bruker 600 MHz (^1^H Larmor frequency) AVANCE III solution NMR spectrometer, equipped with a SampleJet automatic sample loading system, a 5 mm triple resonance (^1^H, ^15^N, ^13^C), liquid-helium-cooled TCI NMR cryoprobe, and Topspin software (Bruker version 3.6). 1D ^1^H NMR spectra acquisition was performed using the Bruker-supplied excitation sculpting (ES)-based ‘zgesgp’ pulse sequence, and NMR spectra were recorded with 256 scans and a ^1^H spectral window of 7211.538 Hz. Free induction decays (FIDs) were collected with 64K data points and a dwell time interval of 69.33 µsec, amounting to a data acquisition time of 4.54 s. Relaxation delay (D1) times between acquisitions were set to 2 s, resulting in an overall 6.5 s delay between scans. DSS chemical shift referencing and phase correction of 1D ^1^H NMR spectra were conducted using Topspin software (Bruker version 3.6).

For verification of Chenomx-annotated metabolites, 2D ^1^H-^1^H total correlation spectroscopy (TOCSY) spectra were acquired for representative samples using the Bruker-supplied ‘mlevphpr.2/mlevgpph19’ pulse sequences (256 × 2048 data points, 2 s relaxation delay, 32 transients per FID, ^1^H spectral window of 6602.11 Hz, 80 ms TOCSY spin lock mixing period). 2D ^1^H-^1^H TOCSY spectra were processed using Topspin software (Bruker version 3.6).

**S2. NMR Data Analysis**

Further processing of 1D ^1^H NMR spectra and metabolite profiling analyses were conducted using the Chenomx NMR Suite software (version 8.1; Chenomx Inc., Edmonton, Alberta, Canada). Baseline correction of NMR spectra following import of preprocessed ‘1r’ NMR spectral files into Chenomx software was performed using the automatic cubic spline function in Chenomx, and subsequent manual breakpoint adjustment to obtain a flat, well-defined baseline, following recommendations from Chenomx application notes and previously reported methods (Emwas et al. 2018). ^1^H chemical shifts were referenced to the 0.0 ppm DSS signal, and the ^1^H NMR signals arising from imidazole were used to correct for small chemical shift changes due to slight variations in sample pH. Metabolite identification and quantification were performed by fitting the 1D ^1^H spectral splitting patterns, chemical shifts, and spectral intensities to reference spectral patterns of small molecules using the Chenomx small molecule spectral database for 600 MHz (^1^H Larmor frequency) magnetic field strength NMR, and manually peak-based fits, where adjustments were made to achieve optimal spectral pattern fits for compound peak cluster location and intensity. An internal (0.25 mM DSS) standard was used for metabolite quantitation. Although pulse sequences utilizing the “ZGESGP” pulse sequence scheme suppress proton signals around the water region to a greater extent than the NOESYPR pulse sequence (i.e. 1D NOESY with presaturation during relaxation and mixing time), the relative intensities observed for these particular ^1^H signals whose resonance frequencies are close to that of the water ^1^H are largely identical to those seen using the noesypr1d sequence. To adjust for minor differences between 1D ^1^H spectra acquired using ‘zgesgp’ versus ‘noesypr1d’ pulse sequences, we have created our own in-house ‘zgesgp’-acquired 600 MHz metabolite library using pure standards and the ‘Compound Builder’ module of Chenomx NMR Suite program (version 8.1), as described previously in the operational manual.

**Table S2**. ICP-OES parameters, conditions of analysis and results for reference material.

| ICP-OES Agilent 725 |  |
| --- | --- |
| Power (kW) | 1.35 |
| Plasma flow (L/min) | 15.0 |
| Auxiliary flow (L/min) | 1.50 |
| Nebulizer pressure (kPa) | 200 |
| Nebulization system | Cross flow (Groove) |
| Number of Replicates | 5 |

| **Reference material - NIST 1577** | | | | | | | | |
| --- | --- | --- | --- | --- | --- | --- | --- | --- |
| **Ca** | **Cu** | **Fe** | **K** | **Mg** | **Mn** | **Na** | **S** | **Zn** |
| **mg/kg** | **mg/kg** | **mg/kg** | **mg/kg** | **mg/kg** | **mg/kg** | **mg/kg** | **mg/kg** | **mg/kg** |
| 131 | 275.2 | 197.94 | 10230 | 620 | 10.46 | 2033 | 7490 | 181.1 |

**Figure S1.** OPLS-DA model validation of ^1^H NMR data (normal and tumor tissues). (A) The permutation test showing the observed and cross-validated R^2^Y and Q^2^ coefficients based on 2000 permutations of metabolites from two groups of kidney tissue samples with a statistically significant p value < 5E-04 (0/2000). (B) Model overview showing the R^2^X, R^2^Y and Q^2^ coefficients for the groups.

**Figure S2.** (A) Receiver operating curve (ROC) illustrating the performance of the NMR models in distinguishing between tumor and normal tissue using random forest algorithm on five chosen potential metabolite biomarkers. (B) Permutation test based on measure area under ROC curve. The p value based on permutation is < 0.001(0/1000). (C) The average of predicted class probabilities of each sample across the 100 cross-validations. (D) The permutation test with predictive accuracy. The average accuracy based on 100 cross validations is 0.904.

**Table S3.** Mean metabolite concentrations (nmol/g of tissue) for controls vs. kidney cancer tissue extracts on ^1^H NMR data set. Those metabolites highlighted in bold are considered statistically significantly different (p < 0.05; FDR < 0.05; |p(corr)| > 0.5) between controls and kidney cancer serum extracts.

| **No.** | **Name** | **Control** | | **Cancer** | | **p-value** | **q-value (FDR)** | **Fold Change** | **AUC** | **p[1]** | **p(corr)[1]** |
| --- | --- | --- | --- | --- | --- | --- | --- | --- | --- | --- | --- |
|  |  | **Mean** | **SD** | **Mean** | **SD** |  |  |  |  |  |  |
| 1 | 3-Hydroxybutyrate | 0.1322 | 0.1472 | 0.1120 | 0.1294 | 2.5E-01 | 3.4E-01 | 1.2 | 0.54 | -0.269 | -0.119 |
| 2 | Acetate | 0.4250 | 0.7211 | 0.2514 | 0.3915 | 4.7E-07 | 2.2E-06 | 1.7 | 0.72 | -0.752 | -0.334 |
| 3 | Alanine | 1.2383 | 0.7038 | 0.8812 | 0.6793 | 3.1E-04 | 7.1E-04 | 1.4 | 0.66 | -0.808 | -0.359 |
| 4 | Aspartate | 0.4579 | 0.2593 | 0.4319 | 0.3129 | 5.2E-01 | 6.0E-01 | 1.1 | 0.56 | 0.165 | 0.073 |
| 5 | Betaine | 0.4929 | 0.5998 | 0.3756 | 0.4100 | 1.5E-02 | 2.6E-02 | 1.3 | 0.59 | -0.447 | -0.198 |
| 6 | Carnitine | 0.2194 | 0.1378 | 0.2595 | 0.1534 | 4.1E-01 | 4.8E-01 | 0.8 | 0.59 | 0.255 | 0.113 |
| 7 | Choline | 0.7286 | 0.8538 | 0.8543 | 1.0715 | 7.2E-01 | 7.5E-01 | 0.9 | 0.53 | -0.102 | -0.045 |
| 8 | Citrate | 0.0840 | 0.0839 | 0.1170 | 0.0960 | 4.0E-01 | 4.8E-01 | 0.7 | 0.61 | -0.245 | -0.109 |
| 9 | Creatine | 0.1912 | 0.2076 | 0.6132 | 0.9385 | 6.1E-06 | 2.0E-05 | 0.3 | 0.76 | 1.074 | 0.477 |
| 10 | Creatinine | 0.2208 | 0.1474 | 0.3090 | 0.2882 | 3.7E-01 | 4.6E-01 | 0.7 | 0.55 | 0.245 | 0.109 |
| 11 | Ethanolamine | 0.6967 | 0.8254 | 0.4104 | 0.6385 | 3.2E-10 | 5.2E-09 | 1.7 | 0.76 | -0.920 | -0.409 |
| 12 | Formate | 0.0272 | 0.0242 | 0.0217 | 0.0206 | 2.0E-02 | 3.4E-02 | 1.3 | 0.61 | -0.588 | -0.261 |
| **13** | **Fumarate** | **0.1020** | **0.0480** | **0.0327** | **0.0321** | **4.1E-11** | **9.8E-10** | **3.1** | **0.91** | **-1.535** | **-0.681** |
| 14 | Glucose | 0.5729 | 0.4243 | 0.8735 | 1.1097 | 3.7E-01 | 4.6E-01 | 0.7 | 0.57 | -0.226 | -0.100 |
| 15 | Glutamate | 1.5943 | 0.6674 | 2.2514 | 1.5503 | 1.3E-01 | 1.9E-01 | 0.7 | 0.63 | -0.091 | -0.040 |
| 16 | Glycerol | 1.0638 | 1.6945 | 0.5606 | 0.8100 | 1.7E-06 | 6.4E-06 | 1.9 | 0.66 | -1.004 | -0.446 |
| 17 | Glycine | 1.3264 | 0.6160 | 1.3884 | 0.8480 | 6.4E-01 | 6.9E-01 | 1.0 | 0.51 | -0.157 | -0.070 |
| 18 | Hippurate | 0.0230 | 0.0544 | 0.0165 | 0.0537 | 7.2E-01 | 7.5E-01 | 1.4 | 0.52 | -0.112 | -0.050 |
| 19 | Histidine | 0.0781 | 0.0579 | 0.1012 | 0.2726 | 3.6E-01 | 4.6E-01 | 0.8 | 0.57 | -0.151 | -0.067 |
| 20 | Hypoxanthine | 0.4062 | 0.2099 | 0.2293 | 0.1933 | 8.8E-08 | 5.3E-07 | 1.8 | 0.77 | -0.802 | -0.356 |
| 21 | IMP | 0.0159 | 0.0190 | 0.0281 | 0.0375 | 6.6E-02 | 1.0E-01 | 0.6 | 0.62 | 0.504 | 0.224 |
| 22 | Inosine | 0.2353 | 0.1728 | 0.2293 | 0.1822 | 1.1E-01 | 1.7E-01 | 1.0 | 0.54 | -0.175 | -0.078 |
| 23 | Isoleucine | 0.0934 | 0.0380 | 0.0589 | 0.0389 | 5.0E-07 | 2.2E-06 | 1.6 | 0.79 | -1.092 | -0.485 |
| 24 | Lactate | 7.3129 | 4.0356 | 9.9277 | 5.1184 | 2.8E-03 | 5.7E-03 | 0.7 | 0.68 | 0.572 | 0.254 |
| **25** | **Leucine** | **0.2672** | **0.1054** | **0.1208** | **0.1068** | **2.6E-11** | **9.8E-10** | **2.2** | **0.90** | **-1.333** | **-0.592** |
| 26 | Methanol | 0.5791 | 0.5770 | 0.4115 | 0.3921 | 5.2E-03 | 9.9E-03 | 1.4 | 0.61 | -0.523 | -0.232 |
| 27 | Methionine | 0.1457 | 0.0713 | 0.1615 | 0.1112 | 9.6E-01 | 9.6E-01 | 0.9 | 0.52 | 0.056 | 0.025 |
| 28 | *myo*-Inositol | 1.6132 | 1.0402 | 1.1294 | 1.2237 | 9.0E-05 | 2.3E-04 | 1.4 | 0.73 | -0.825 | -0.366 |
| 29 | NAD | 0.0651 | 0.0540 | 0.0600 | 0.0710 | 1.3E-01 | 1.9E-01 | 1.1 | 0.58 | -0.070 | -0.031 |
| 30 | Nicotinurate | 0.0525 | 0.0518 | 0.0509 | 0.0455 | 9.1E-01 | 9.3E-01 | 1.0 | 0.50 | 0.107 | 0.047 |
| **31** | **Phenylalanine** | **0.1251** | **0.0494** | **0.0682** | **0.0669** | **6.1E-06** | **2.0E-05** | **1.8** | **0.78** | **-1.129** | **-0.501** |
| 32 | Proline | 0.3379 | 0.2122 | 0.2615 | 0.2402 | 5.5E-03 | 1.0E-02 | 1.3 | 0.65 | -0.534 | -0.237 |
| 33 | Propylene glycol | 0.0326 | 0.0354 | 0.0346 | 0.0965 | 7.8E-06 | 2.3E-05 | 0.9 | 0.65 | -0.872 | -0.387 |
| 34 | Pyroglutamate | 0.4554 | 0.3248 | 0.3126 | 0.2421 | 2.0E-03 | 4.2E-03 | 1.5 | 0.65 | -0.683 | -0.303 |
| 35 | Pyruvate | 0.1832 | 0.0695 | 0.2531 | 0.1558 | 7.3E-02 | 1.1E-01 | 0.7 | 0.65 | 0.274 | 0.121 |
| **36** | **Sarcosine** | **0.1062** | **0.0696** | **0.0277** | **0.0451** | **7.7E-09** | **8.0E-08** | **3.8** | **0.89** | **-1.389** | **-0.617** |
| 37 | *sn*-Glycero-3-phosphocholine | 0.2095 | 0.1910 | 0.4610 | 0.4055 | 8.3E-06 | 2.3E-05 | 0.5 | 0.75 | 1.031 | 0.458 |
| 38 | Succinate | 0.0415 | 0.0885 | 0.1698 | 0.2744 | 6.7E-05 | 1.8E-04 | 0.2 | 0.72 | 0.835 | 0.371 |
| 39 | Taurine | 2.0666 | 0.9842 | 2.1207 | 1.3472 | 3.5E-01 | 4.6E-01 | 1.0 | 0.50 | -0.200 | -0.089 |
| 40 | Threonine | 0.5445 | 0.2390 | 0.4142 | 0.1797 | 7.6E-04 | 1.7E-03 | 1.3 | 0.65 | -0.684 | -0.304 |
| 41 | Trimethylamine | 0.2423 | 0.2048 | 0.1808 | 0.1653 | 2.2E-02 | 3.6E-02 | 1.3 | 0.56 | -0.317 | -0.141 |
| 42 | Tryptophan | 0.0426 | 0.0194 | 0.0172 | 0.0147 | 3.9E-08 | 2.6E-07 | 2.5 | 0.86 | -1.298 | -0.576 |
| 43 | Tyrosine | 0.1493 | 0.0729 | 0.0744 | 0.0636 | 3.5E-07 | 1.9E-06 | 2.0 | 0.82 | -0.957 | -0.425 |
| 44 | Uracil | 0.0647 | 0.0447 | 0.0435 | 0.0439 | 3.1E-04 | 7.1E-04 | 1.5 | 0.69 | -0.485 | -0.215 |
| 45 | Urea | 5.9407 | 2.5319 | 4.1076 | 2.3725 | 1.5E-08 | 1.2E-07 | 1.4 | 0.75 | -0.937 | -0.416 |
| 46 | Uridine | 0.0900 | 0.0735 | 0.0834 | 0.0686 | 6.2E-01 | 6.9E-01 | 1.1 | 0.53 | 0.375 | 0.167 |
| 47 | Valine | 0.2260 | 0.0926 | 0.1509 | 0.0907 | 1.4E-06 | 5.7E-06 | 1.5 | 0.77 | -1.015 | -0.450 |
| 48 | Xanthine | 0.1263 | 0.0584 | 0.0528 | 0.0505 | 8.3E-09 | 8.0E-08 | 2.4 | 0.85 | -1.051 | -0.466 |

**Figure S3.** OPLS-DA model validation of ^1^H NMR data set (benign and malignant tumor). (A) The permutation test showing the observed and cross-validated R^2^Y and Q^2^ coefficients based on 2000 permutations of metabolites from the two groups of kidney tissue samples from patients with benign and malignant type of cancer with a statistically significant p value < 5E-04 (0/2000). (B) Model overview showing the R^2^X, R^2^Y and Q^2^ coefficients for the groups.

**Figure S4.** (A) Receiver operating curve (ROC) illustrating the performance of the NMR models in distinguishing between benign and malignant tumor using random forest algorithm on two chosen potential metabolite biomarkers. (B) Permutation test based on measure area under ROC curve. The p value based on permutation is < 0.003 (0/1000). (C) The average of predicted class probabilities of each sample across the 100 cross-validations. (D) The permutation test with predictive accuracy. The average accuracy based on 100 cross validations is 0.848.

**Table S4.** Mean chemical elements concentrations (mg/kg) for controls and kidney cancer tissue based on ICP-OES data set. Those variables highlighted in bold are considered statistically significantly different (p<0.05; FDR < 0.05; |p(corr)|>0.5) between normal and kidney tumor tissue extracts.

| **No.** | **Chemical elements** | **Control** | | **Cancer** | | **p-value** | **q-value (FDR)** | **Fold Change** | **P(corr)** | **p[1]** | **AUC** |
| --- | --- | --- | --- | --- | --- | --- | --- | --- | --- | --- | --- |
|  |  | **Mean** | **SD** | **Mean** | **SD** |  |  |  |  |  |  |
| 1 | Ca | 10460 | 50897 | 1002 | 725 | 6.62E-01 | 7.4E-01 | 0.6 | 0.015 | 0.021 | 0.53 |
| 2 | Cu | 15 | 4 | 11 | 11 | 1.04E-02 | 1.7E-02 | 1.0 | -0.247 | -0.330 | 0.71 |
| 3 | Fe | 378 | 291 | 827 | 992 | 1.04E-02 | 1.7E-02 | 0.4 | 0.324 | 0.433 | 0.71 |
| 4 | K | 8902 | 2445 | 8503 | 3981 | 3.88E-03 | 9.7E-03 | 0.8 | 0.175 | 0.234 | 0.74 |
| 5 | Mg | 222 | 648 | 41 | 96 | 6.11E-01 | 7.4E-01 | 2.3 | -0.111 | -0.148 | 0.53 |
| 6 | Mn | 5 | 3 | 3 | 3 | 2.11E-01 | 3.0E-01 | 1.2 | -0.157 | -0.209 | 0.62 |
| **7** | **Na** | **10395** | **2391** | **7467** | **5373** | **2.47E-03** | **8.2E-03** | **1.2** | **-0.510** | **-0.682** | **0.73** |
| 8 | P | 11406 | 19325 | 6156 | 3263 | 8.97E-01 | 9.0E-01 | 1.0 | 0.059 | 0.079 | 0.54 |
| **9** | **S** | **8090** | **1340** | **5869** | **2597** | **2.59E-05** | **1.3E-04** | **1.2** | **-0.750** | **-1.003** | **0.84** |
| **10** | **Zn** | **196** | **81** | **64** | **51** | **7.99E-06** | **8.0E-05** | **2.4** | **-0.866** | **-1.158** | **0.94** |

**Figure S5.** OPLS-DA model validation of ICP-OES data set (normal and tumor tissues). (A) The the permutation test based on 2000 permutations with a statistically significant p value < 5E-04 (0/2000). (B) Model overview showing the R^2^X, R^2^Y and Q^2^ coefficients for the groups.

**Figure S6.** (A) Receiver operating curve (ROC) illustrating the performance of the ICP OES models in distinguishing between tumor and normal tissue using random forest algorithm on three chosen elements (zinc, sulfur and sodium). (B) Permutation test based on measure area under ROC curve. The p value based on permutation is <0.001 (0/1000). (C) The average of predicted class probabilities of each sample across the 100 cross-validations. (D) The permutation test with predictive accuracy. The average accuracy based on 100 cross validations is 0.899.

**Figure S7.** Tissue metabolite profiles for non-polar (A, B) and polar (C, D) extracts of tissue kidney cancer group based on ^109^Ag NPET LDI MS. (A, C) 3D PCA and (B, D) OPLS-DA scores plots generated from the MS data of the tumor tissue (green) and adjacent control tissue (red) samples.

**Figure S8**. OPLS-DA details of the ^109^AgNPET LDI MS data from non-polar (A-C) and polar (D-F) extracts of kidney cancer tissue. (A, D) The OPLS-DA loading S-plot showing the distribution patterns of metabolites to the differences between cancer and normal tissue samples. (B, E) The permutation test based on 2000 permutations of metabolites of kidney tissue samples with a statistically significant p value < 5E-04 (0/2000). (C, F) Model overview showing the R^2^X, R^2^Y and Q^2^ coefficients for the groups

**Table S5.** Mean feature abundance for normal *vs*. cancer kidney tissue non-polar extracts based on LDI MS data set. Those variables highlighted in bold are considered statistically significantly different (p<0.05; FDR < 0.05; |p(corr)|>0.5) between normal and kidney tumor tissue extracts.

| **No.** | ***m/z*** | **Control** | | **Cancer** | | **p-value** | **q-value (FDR)** | **Fold Change** | **p(corr)** | **p[1]** | **AUC** |
| --- | --- | --- | --- | --- | --- | --- | --- | --- | --- | --- | --- |
|  |  | Mean | SD | Mean | SD |  |  |  |  |  |  |
| 1 | 81.084 | 34770 | 17197 | 36320 | 24603 | 1.18E-02 | 3.17E-02 | 1.2 | 0.133 | 0.638 | 0.743 |
| 2 | 82.085 | 3275 | 1845 | 3279 | 2248 | 3.24E-03 | 1.29E-02 | 1.2 | -0.049 | -0.235 | 0.743 |
| 3 | 83.100 | 27958 | 14864 | 30046 | 18499 | 2.68E-05 | 5.70E-04 | 1.3 | -0.255 | -1.228 | 0.826 |
| 4 | 91.068 | 15436 | 7165 | 16043 | 9558 | 6.25E-06 | 3.59E-04 | 1.3 | -0.246 | -1.183 | 0.794 |
| 5 | 93.087 | 15048 | 7371 | 17172 | 10842 | 8.97E-06 | 3.96E-04 | 1.4 | -0.28 | -1.347 | 0.801 |
| 6 | 95.103 | 33442 | 16142 | 45237 | 31564 | 4.13E-06 | 3.59E-04 | 1.7 | -0.372 | -1.793 | 0.83 |
| 7 | 96.101 | 3392 | 1876 | 3987 | 2973 | 1.27E-04 | 1.49E-03 | 1.6 | -0.161 | -0.776 | 0.8 |
| 8 | 97.119 | 13731 | 7502 | 14180 | 8836 | 3.60E-05 | 6.67E-04 | 1.2 | -0.25 | -1.205 | 0.803 |
| 9 | 105.085 | 11817 | 5999 | 13287 | 9094 | 1.46E-05 | 4.94E-04 | 1.4 | -0.079 | -0.38 | 0.82 |
| 10 | 109.114 | 23043 | 15649 | 29187 | 24259 | 2.84E-04 | 2.33E-03 | 1.5 | -0.142 | -0.682 | 0.764 |
| **11** | **121.122** | **90** | **539** | **8248** | **11562** | **1.34E-05** | **4.80E-04** | **42.3** | **-0.586** | **-2.824** | **0.775** |
| 12 | 123.137 | 5764 | 3146 | 6330 | 4339 | 5.93E-05 | 8.72E-04 | 1.4 | -0.124 | -0.598 | 0.773 |
| **13** | **147.137** | **1492** | **3604** | **7446** | **9257** | **2.36E-05** | **5.70E-04** | **6.9** | **-0.524** | **-2.525** | **0.773** |
| 14 | 149.151 | 833 | 1879 | 2913 | 3776 | 2.26E-04 | 2.03E-03 | 4.5 | -0.462 | -2.226 | 0.74 |
| 15 | 157.955 | 15496 | 8218 | 10878 | 7197 | 1.04E-04 | 1.30E-03 | 0.8 | 0.253 | 1.217 | 0.759 |
| **16** | **161.153** | **574** | **1542** | **4378** | **5786** | **7.84E-06** | **3.75E-04** | **8.3** | **-0.501** | **-2.411** | **0.768** |
| 17 | 173.951 | 9997 | 5517 | 7009 | 4909 | 2.26E-05 | 5.70E-04 | 0.8 | 0.139 | 0.668 | 0.744 |
| 18 | 182.871 | 8219 | 4291 | 6129 | 3751 | 5.03E-05 | 8.02E-04 | 0.9 | 0.338 | 1.629 | 0.754 |
| 19 | 186.870 | 10086 | 5164 | 7198 | 4356 | 2.41E-07 | 6.92E-05 | 0.8 | 0.405 | 1.951 | 0.848 |
| 20 | 206.845 | 7753 | 4480 | 5729 | 3982 | 1.04E-04 | 1.30E-03 | 0.8 | 0.22 | 1.062 | 0.751 |
| 21 | 210.856 | 8862 | 5906 | 6445 | 4487 | 4.44E-05 | 7.28E-04 | 0.9 | 0.038 | 0.184 | 0.757 |
| 22 | 220.834 | 5291 | 3277 | 3778 | 2785 | 2.46E-05 | 5.70E-04 | 0.8 | 0.194 | 0.935 | 0.771 |
| 23 | 223.840 | 4382 | 2741 | 2601 | 2271 | 3.17E-04 | 2.57E-03 | 0.7 | 0.204 | 0.982 | 0.782 |
| 24 | 224.840 | 4596 | 2805 | 3201 | 2454 | 2.73E-04 | 2.33E-03 | 0.8 | 0.234 | 1.129 | 0.752 |
| 25 | 227.967 | 3232 | 2135 | 1810 | 1713 | 5.97E-06 | 3.59E-04 | 0.7 | 0.319 | 1.535 | 0.755 |
| 26 | 228.969 | 3991 | 2744 | 2692 | 2135 | 5.89E-04 | 3.93E-03 | 0.8 | 0.056 | 0.268 | 0.742 |
| 27 | 229.979 | 3807 | 2433 | 2475 | 1810 | 1.00E-04 | 1.30E-03 | 0.8 | 0.147 | 0.71 | 0.748 |
| 28 | 236.130 | 8688 | 5293 | 5607 | 3612 | 4.08E-05 | 7.10E-04 | 0.8 | 0.371 | 1.785 | 0.792 |
| 29 | 241.083 | 3934 | 2755 | 2285 | 1993 | 2.68E-05 | 5.70E-04 | 0.7 | 0.288 | 1.388 | 0.778 |
| **30** | **243.824** | **19767** | **12042** | **12878** | **9762** | **1.03E-05** | **4.21E-04** | **0.8** | **0.518** | **2.495** | **0.768** |
| 31 | 244.964 | 4875 | 3431 | 2434 | 2594 | 4.75E-06 | 3.59E-04 | 0.6 | 0.256 | 1.233 | 0.771 |
| 32 | 263.778 | 3587 | 2168 | 2358 | 1903 | 5.46E-05 | 8.25E-04 | 0.7 | 0.259 | 1.248 | 0.766 |
| 33 | 267.796 | 3839 | 2428 | 2575 | 1897 | 2.18E-04 | 1.99E-03 | 0.8 | 0.189 | 0.909 | 0.742 |
| 34 | 272.775 | 5145 | 3175 | 3549 | 2521 | 1.51E-07 | 6.92E-05 | 0.8 | 0.325 | 1.566 | 0.816 |
| 35 | 280.771 | 4806 | 3180 | 3158 | 2226 | 2.47E-06 | 3.54E-04 | 0.8 | 0.177 | 0.854 | 0.765 |
| 36 | 285.772 | 2390 | 1735 | 1396 | 1301 | 1.27E-04 | 1.49E-03 | 0.7 | 0.236 | 1.136 | 0.741 |
| 37 | 286.955 | 5639 | 4997 | 3322 | 2893 | 1.67E-05 | 5.04E-04 | 0.7 | 0.281 | 1.352 | 0.77 |
| 38 | 287.765 | 3408 | 2212 | 2291 | 1688 | 2.46E-05 | 5.70E-04 | 0.8 | 0.351 | 1.688 | 0.746 |
| 39 | 288.765 | 3150 | 2228 | 2162 | 1632 | 1.03E-03 | 5.86E-03 | 0.8 | 0.169 | 0.816 | 0.753 |
| 40 | 294.773 | 1982 | 1641 | 832 | 1161 | 3.27E-06 | 3.59E-04 | 0.5 | 0.438 | 2.11 | 0.756 |
| 41 | 296.774 | 2899 | 2130 | 1668 | 1472 | 6.97E-05 | 1.00E-03 | 0.7 | 0.352 | 1.695 | 0.79 |
| 42 | 325.131 | 3113 | 2529 | 1505 | 1421 | 3.04E-05 | 5.82E-04 | 0.6 | 0.319 | 1.536 | 0.784 |
| 43 | 331.172 | 5008 | 4622 | 7834 | 7264 | 2.63E-04 | 2.29E-03 | 1.9 | -0.298 | -1.435 | 0.754 |
| **44** | **331.568** | **1118** | **2601** | **2824** | **2971** | **5.46E-05** | **8.25E-04** | **2.8** | **-0.522** | **-2.513** | **0.751** |
| 45 | 332.176 | 1853 | 1948 | 2724 | 2678 | 1.67E-04 | 1.65E-03 | 1.9 | -0.269 | -1.297 | 0.751 |
| 46 | 333.188 | 7409 | 9268 | 12596 | 11384 | 1.54E-04 | 1.64E-03 | 2.1 | -0.438 | -2.109 | 0.75 |
| 47 | 334.191 | 3017 | 3633 | 5227 | 4899 | 3.92E-05 | 7.03E-04 | 2.2 | -0.393 | -1.892 | 0.775 |
| 48 | 337.195 | 1579 | 2498 | 2130 | 1896 | 1.00E-04 | 1.30E-03 | 1.8 | -0.388 | -1.868 | 0.75 |
| 49 | 343.696 | 2071 | 1529 | 1083 | 1135 | 1.12E-05 | 4.29E-04 | 0.6 | 0.321 | 1.548 | 0.796 |
| 50 | 359.189 | 249 | 658 | 979 | 1158 | 2.68E-05 | 5.70E-04 | 4.6 | -0.479 | -2.305 | 0.774 |
| **51** | **361.208** | **120** | **397** | **1085** | **1429** | **7.84E-06** | **3.75E-04** | **8.7** | **-0.569** | **-2.741** | **0.789** |
| 52 | 391.157 | 4758 | 5254 | 10877 | 12780 | 2.36E-05 | 5.70E-04 | 3.5 | -0.379 | -1.827 | 0.772 |
| 53 | 392.169 | 572 | 1268 | 1837 | 2519 | 1.13E-04 | 1.38E-03 | 5.6 | -0.466 | -2.246 | 0.747 |
| 54 | 538.373 | 22353 | 21881 | 11592 | 14070 | 1.32E-04 | 1.52E-03 | 0.6 | 0.318 | 1.531 | 0.742 |
| 55 | 539.373 | 6113 | 6567 | 3144 | 3821 | 1.80E-04 | 1.75E-03 | 0.6 | 0.21 | 1.011 | 0.742 |
| 56 | 553.343 | 9567 | 7837 | 5369 | 7467 | 2.92E-05 | 5.82E-04 | 0.6 | 0.288 | 1.387 | 0.757 |
| 57 | 576.478 | 1710 | 1399 | 972 | 950 | 3.96E-04 | 3.03E-03 | 0.6 | 0.309 | 1.49 | 0.748 |
| 58 | 623.283 | 46171 | 38894 | 25200 | 24792 | 1.32E-06 | 2.53E-04 | 0.6 | 0.361 | 1.738 | 0.764 |
| 59 | 624.293 | 14287 | 14153 | 7063 | 6730 | 3.04E-05 | 5.82E-04 | 0.6 | 0.179 | 0.862 | 0.75 |
| 60 | 627.467 | 1262 | 1368 | 539 | 680 | 1.67E-05 | 5.04E-04 | 0.5 | 0.403 | 1.939 | 0.75 |

**Table S6.** Mean metabolite abundance for normal *vs*. cancer kidney tissue polar extracts based on LDI MS data set. Those variables highlighted in bold are considered statistically significantly different (p<0.05; FDR < 0.05; |p(corr)|>0.5) between normal and kidney tumor tissue extracts.

| No. | *m/z* | Control | | Cancer | | p-value | q-value (FDR) | Fold Change | p(corr) | p[1] | AUC |
| --- | --- | --- | --- | --- | --- | --- | --- | --- | --- | --- | --- |
|  |  | Mean | SD | Mean | SD |  |  |  |  |  |  |
| 1 | 94.952 | 1222 | 564 | 2024 | 1157 | 1.90E-05 | 3.97E-04 | 1.8 | -0.342 | -1.415 | 0.779 |
| 2 | 95.984 | 1340 | 578 | 1836 | 686 | 4.58E-04 | 3.76E-03 | 1.2 | -0.256 | -1.059 | 0.744 |
| **3** | **120.940** | **6143** | **2149** | **9135** | **5056** | **9.39E-06** | **2.54E-04** | **1.7** | **-0.516** | **-2.134** | **0.758** |
| 4 | 136.953 | 1861 | 2021 | 3125 | 2429 | 4.45E-07 | 9.58E-05 | 0.6 | -0.332 | -1.372 | 0.798 |
| **5** | **141.991** | **348** | **586** | **1478** | **1005** | **8.95E-07** | **9.58E-05** | **4.0** | **-0.568** | **-2.348** | **0.836** |
| **6** | **142.057** | **2545** | **1761** | **982** | **1453** | **6.54E-06** | **2.31E-04** | **0.4** | **0.572** | **2.367** | **0.750** |
| 7 | 147.898 | 701 | 737 | 1317 | 1533 | 1.07E-05 | 2.74E-04 | 2.0 | -0.312 | -1.290 | 0.756 |
| 8 | 148.957 | 2475 | 1210 | 3780 | 2939 | 1.54E-04 | 1.61E-03 | 1.0 | -0.175 | -0.726 | 0.790 |
| 9 | 159.042 | 18507 | 10948 | 8863 | 6601 | 1.20E-06 | 9.58E-05 | 0.6 | 0.418 | 1.729 | 0.753 |
| 10 | 163.968 | 306 | 728 | 1231 | 1490 | 1.68E-06 | 9.68E-05 | 3.6 | -0.487 | -2.014 | 0.799 |
| 11 | 169.989 | 21013 | 17250 | 8924 | 12943 | 1.26E-06 | 9.58E-05 | 0.5 | 0.494 | 2.043 | 0.742 |
| 12 | 170.028 | 1518 | 4311 | 9777 | 17086 | 4.75E-06 | 1.99E-04 | 6.5 | -0.498 | -2.059 | 0.744 |
| 13 | 175.013 | 11066 | 5991 | 5249 | 3380 | 9.87E-07 | 9.58E-05 | 0.5 | 0.441 | 1.824 | 0.771 |
| **14** | **181.015** | **4823** | **3277** | **1421** | **2417** | **2.35E-06** | **1.08E-04** | **0.3** | **0.569** | **2.355** | **0.748** |
| 15 | 223.000 | 1665 | 1221 | 2229 | 1291 | 1.27E-04 | 1.50E-03 | 1.1 | -0.311 | -1.286 | 0.742 |
| 16 | 257.975 | 2426 | 1899 | 1023 | 850 | 8.58E-06 | 2.54E-04 | 0.5 | 0.429 | 1.776 | 0.775 |
| 17 | 325.050 | 1083 | 1344 | 477 | 1290 | 5.97E-06 | 2.29E-04 | 0.5 | 0.490 | 2.027 | 0.746 |
| 18 | 365.146 | 4483 | 8163 | 7794 | 10574 | 1.80E-04 | 1.73E-03 | 0.3 | -0.345 | -1.427 | 0.748 |
| 19 | 462.749 | 1454 | 1993 | 533 | 568 | 7.56E-05 | 1.12E-03 | 0.4 | 0.420 | 1.737 | 0.752 |
| **20** | **491.781** | **321** | **374** | **64** | **111** | **3.31E-05** | **6.09E-04** | **0.3** | **0.560** | **2.318** | **0.747** |

**Figure S9.** ROC curve analysis for potential biomarkers predicted by classical univariate analysis of data from ^109^AgNPET LDI MS for non-polar (A-F) and polar (G-K) extracts of studied tissues. The left-hand side of each panel indicates ROC curve for a particular metabolite with 95% confidence interval (shadowed) and the solid red dot indicates the optimal cut-off associated with sensitivity and specificity values. The right-hand side of each panel depicts the distribution of metabolite level values observed in control and kidney cancer tissue samples. The horizontal red line in the graphs indicates the cut-off point.

**Figure S10.** ROC curve analysis for non-polar (A, B) and polar (C, D) extracts of tissue kidney cancer group based on ^109^Ag NPET LDI MS. (A, B) A combination metabolites model calculated from the logistic regression analysis. (B, D) Metabolites with the highest ability to discriminate tumor kidney tissue against controls.

**Figure S11.** (A) ROC curves illustrating the performance of the ^109^AgNPET LDI MS model in distinguishing between tumor and normal tissue using random forest algorithm on five selected features from polar extracts of studied tissues (B) Permutation test based on measure area under ROC curve. The p value based on permutation is < 0.001 (0/1000). (C) The average of predicted class probabilities of each sample across the 100 cross-validations. (D) The permutation test with predictive accuracy. The average accuracy based on 100 cross validations is 0.807

**Figure S12.** (A) ROC curves illustrating the performance of the ^109^AgNPET LDI MS model in distinguishing between tumor and normal tissue using random forest algorithm on six selected features from non-polar extracts of studied tissues (B) Permutation test based on measure area under ROC curve. The p value based on permutation is < 0.001 (0/1000). (C) The average of predicted class probabilities of each sample across the 100 cross-validations. (D) The permutation test with predictive accuracy. The average accuracy based on 100 cross validations is 0.733.

**Figure S13.** Metabolite profiles based on ^1^H NMR spectra of tissue extracts from 30 male and 19 female patients with kidney cancer (A) 2D PCA and (B) 3D PCA scores plots generated from the ^1^H NMR data of the female tumor tissue (red), adjacent control female tissue (green), male tumor tissue (violet) and adjacent control female tissue (sky blue) samples. (C-E) Box and whisker plots reflective of the normalized concentration of three selected metabolite in the tissue extracts of male and female patients. The red, green, dark blue and light blue circles indicate female, female controls, male and male controls patients, respectively.

**Figure S14.** Metabolite profiles based on ^1^H NMR spectra of tissue extracts of 11 patients under age 60 and 37 over age 60 with kidney cancer (A) 2D PCA and (B) 3D PCA scores plots generated from the ^1^H NMR data of the tumor tissue from patients under age 60 (red), adjacent control tissue of patients under age 60 (green), tumor tissue from patients over age 60 (violet) and adjacent control tissue of patients over age 60 (sky blue) samples. (C-E) Box and whisker plots reflective of the normalized concentration of three selected metabolite in the different. The red, green, dark blue and light blue circles indicate age < 60, age < 60 controls, age > 60 and age > 60 controls respectively.

**Figure S15.** Elemental profiles based on ICP-OES data of tissue extracts from 30 male and 19 female patients with kidney cancer (A) 2D PCA and (B) 3D PCA scores plots generated from the ICP-OES data of the female tumor tissue (red), adjacent control female tissue (green), male tumor tissue (violet) and adjacent control female tissue (sky blue) samples. (C-E) Box and whisker plots reflective of the normalized concentration of two selected elements in the tissue extracts of male and female patients. The red, green, dark blue and light blue circles indicate female, female controls, male and male controls patients, respectively.

**Figure S16.** Elemental profiles based on ICP-OES data of tissue extracts of 11 patients under age 60 and 37 over age 60 with kidney cancer (A) 2D PCA and (B) 3D PCA scores plots generated from the ICP-OES data of the tumor tissue from patients under age 60 (red), adjacent control tissue of patients under age 60 (green), tumor tissue from patients over age 60 (violet) and adjacent control tissue of patients over age 60 (sky blue) samples. (C-E) Box and whisker plots reflective of the normalized concentration of two selected elements in the tissue extracts of patients under and over age 60. The red, green, dark blue and light blue circles indicate age < 60, age < 60 controls, age > 60 and age > 60 controls respectively.

**Figure S17.** Metabolite profiles based on ^109^AgNPET LDI MS data of tissue extracts from 30 male and 19 female patients with kidney cancer (A, C) 2D PCA scores plots generated from the MS data of polar (A) and non-polar extracts (C) of studied female tumor tissue (red), adjacent control female tissue (green), male tumor tissue (violet) and adjacent control female tissue (sky blue) samples. (B, D) Box and whisker plots reflective of the normalized level of selected features in the tissue extracts of male and female patients from the MS data of polar (B) and non-polar extracts (D). The red, green, dark blue and light blue circles indicate female, female controls, male and male controls patients, respectively.

**Figure S18.** Metabolite profiles based on ^1^H NMR spectra of tissue extracts of 11 patients under age 60 and 37 over age 60 with kidney cancer (A, C) 2D PCA scores plots generated from the MS data of polar (A) and non-polar extracts (C) of the tumor tissue from patients under age 60 (red), adjacent control tissue of patients under age 60 (green), tumor tissue from patients over age 60 (violet) and adjacent control tissue of patients over age 60 (sky blue) samples. (B, D) Box and whisker plots reflective of the normalized level of selected features in the tissue extracts of patients under and over age 60 from the MS data of polar (B) and non-polar extracts (D). The red, green, dark blue and light blue circles indicate age < 60, age < 60 controls, age > 60 and age > 60 controls respectively.

**Table S7.** Result from Pathway Analysis

| **KEGG pathway** | **Total**^a^ | **Hits**^b^ | **Impact**^c^ | **p-value**^d^ | **Holm p**^e^ | **FDR**^f^ |
| --- | --- | --- | --- | --- | --- | --- |
| Citrate cycle (TCA cycle) | 20 | 1 | 0.030 | 3.50E-11 | 4.20E-10 | 8.40E-11 |
| Tyrosine metabolism | 42 | 1 | 0.025 | 3.50E-11 | 4.20E-10 | 8.39E-11 |
| Alanine, aspartate and glutamate metabolism | 28 | 1 | 0.002 | 3.50E-11 | 4.20E-10 | 8.39E-11 |
| Arginine biosynthesis | 14 | 1 | 0.000 | 3.50E-11 | 4.20E-10 | 8.39E-11 |
| Pyruvate metabolism | 22 | 1 | 0.000 | 3.50E-11 | 4.20E-10 | 8.39E-11 |
| Aminoacyl-tRNA biosynthesis | 48 | 3 | 0.000 | 9.33E-11 | 6.53E-10 | 1.86E-10 |
| Valine, leucine and isoleucine degradation | 40 | 1 | 0.000 | 1.91E-10 | 1.14E-9 | 2.86E-10 |
| Valine, leucine and isoleucine biosynthesis | 8 | 1 | 0.000 | 1.91E-10 | 1.14E-9 | 2.86E-10 |
| Glycine, serine and threonine metabolism | 33 | 1 | 0.091 | 4.00E-9 | 1.60E-8 | 5.33E-9 |
| Tryptophan metabolism | 41 | 1 | 0.143 | 9.23E-9 | 2.77E-8 | 1.11E-8 |
| Phenylalanine, tyrosine and tryptophan biosynthesis | 4 | 1 | 0.500 | 2.17E-7 | 4.33E-7 | 2.17E-7 |
| Phenylalanine metabolism | 10 | 1 | 0.357 | 2.17E-7 | 4.33E-7 | 2.17E-7 |

^a^The total number of compounds in the pathway; ^b^the hits is the actually matched number from the NMR data; ^c^the pathway impact value calculated from pathway topology analysis; ^d^p-value calculated from the enrichment analysis; ^e^p-value adjusted by Holm–Bonferroni method; ^f^p-value adjusted using False Discovery Rate

| **SMPDB pathway** | **Total**^a^ | **Hits**^b^ | **Expected** | **p-value** | **Holm p** | **FDR** |
| --- | --- | --- | --- | --- | --- | --- |
| Tyrosine Metabolism | 72 | 1 | 1.031 | 3.50E-11 | 4.55E-10 | 5.66E-11 |
| Arginine and Proline Metabolism | 53 | 1 | 1.031 | 3.50E-11 | 4.55E-10 | 5.66E-11 |
| Purine Metabolism | 74 | 1 | 1.031 | 3.50E-11 | 4.55E-10 | 5.66E-11 |
| Citric Acid Cycle | 32 | 1 | 1.031 | 3.50E-11 | 4.55E-10 | 5.66E-11 |
| Urea Cycle | 29 | 1 | 1.031 | 3.50E-11 | 4.55E-10 | 5.66E-11 |
| Aspartate Metabolism | 35 | 1 | 1.031 | 3.50E-11 | 4.55E-10 | 5.66E-11 |
| Mitochondrial Electron Transport Chain | 19 | 1 | 1.031 | 3.50E-11 | 4.55E-10 | 5.66E-11 |
| Warburg Effect | 58 | 1 | 1.031 | 3.50E-11 | 4.55E-10 | 5.66E-11 |
| Phenylalanine and Tyrosine Metabolism | 28 | 2 | 1.031 | 3.92E-11 | 4.55E-10 | 5.66E-11 |
| Valine, Leucine and Isoleucine Degradation | 60 | 1 | 1.031 | 1.91E-10 | 7.63E-10 | 2.48E-10 |
| Glycine and Serine Metabolism | 59 | 1 | 1.031 | 4.00E-09 | 1.20E-08 | 4.33E-09 |
| Methionine Metabolism | 43 | 1 | 1.031 | 4.00E-09 | 1.20E-08 | 4.33E-09 |
| Tryptophan Metabolism | 60 | 1 | 1.031 | 9.23E-09 | 1.20E-08 | 9.23E-09 |

**Table S8.** Result from Enrichment Pathway Analysis

^a^The total number of compounds in the pathway; ^b^the hits is the actually matched number from the NMR data; ^c^the pathway impact value calculated from pathway topology analysis; ^d^p-value calculated from the enrichment analysis; ^e^p-value adjusted by Holm–Bonferroni method; ^f^p-value adjusted using False Discovery Rate
